# Remote Ischemic Preconditioning Attenuates Mitochondrial Dysfunction and Ferroptosis of Tubular Epithelial Cells by Inhibiting NOX4-ROS Signaling in Acute Kidney Injury

**DOI:** 10.7150/ijbs.105667

**Published:** 2025-02-26

**Authors:** Wei Wei, Letian Yang, Bo Wang, Lei Tang, Jian Li, Caihong Liu, Yongxiu Huang, Zhuyun Zhang, Dingkun Zhang, Ling Zhang, Liang Ma, Ping Fu, Yuliang Zhao

**Affiliations:** 1 Department of Nephrology, West China Hospital, Sichuan University, Chengdu, China.; 2 Institute of Kidney Diseases, West China Hospital, Sichuan University, Chengdu, China.; 3 Laboratory of Clinical Proteomics and Metabolomics, Institutes for Systems Genetics, Frontiers Science Center for Disease-related Molecular Network, National Clinical Research Center for Geriatrics, West China Hospital, Sichuan University, Chengdu, China.

**Keywords:** remote ischemic preconditioning, NADPH oxidase 4, acute kidney injury, mitochondria, ferroptosis

## Abstract

Acute kidney injury (AKI) is a worldwide clinical burden associated with high morbidity and mortality. Remote ischemic preconditioning (rIPC), a brief nonlethal ischemia and reperfusion (IR) in remote tissues or limbs, has been used in an attempt to protect against AKI, but its underlying signaling pathways has not been elucidated. In the present study, rIPC protected kidney function and pathological injury and mitigated NADPH oxidase 4 (NOX4) upregulation in different AKI models (cisplatin, LPS and IRI). Furthermore, rIPC significantly attenuated mitochondrial dysfunction and ameliorated tubular epithelial ferroptosis during AKI. Mechanistically, in wild-type AKI mice and TCMK-1 cells, rIPC effectively decreased kidney ROS production, preserved mitochondrial dynamics and mitophagy, and ameliorated tubular epithelial ferroptosis. Notably, these protective effects of rIPC were further enhanced by NOX4 knockout or silencing and mitigated by NOX4 overexpression. Our study showed that rIPC may attenuate mitochondrial dysfunction and ferroptosis in tubular epithelial cells in AKI by inhibiting NOX4-ROS signaling. NOX4 might be used as a biomarker for monitoring the biological effects of rIPC to optimize the rIPC protocol and facilitate future translational studies.

## Introduction

Acute kidney injury (AKI) is a worldwide clinical burden associated with high morbidity and mortality and is associated with different etiologies and complex pathophysiologies^1^,^2^. Despite the identification of various biomarkers and prediction algorithms for the early diagnosis of AKI^3^, effective treatments for this disease have not been identified. A deep understanding of the molecular mechanisms involved in AKI is needed for the validation of novel therapeutic options. Remote ischemic preconditioning (rIPC) is a brief nonlethal ischemia and reperfusion (IR) in remote tissues or limbs and has been used in an attempt to protect organ function (heart, brain, kidney, etc.) from subsequent lethal insults^4^, ^5^. rIPC was shown to reduce the occurrence of cardiac surgery-associated AKI, especially among high-risk patients^6^, but its applicability to other forms of AKI, as well as the underlying signaling pathways responsible for its protective effect, remains underinvestigated.

Nicotinamide adenine dinucleotide phosphate (NADPH) oxidase 4 (NOX4), the most abundantly expressed isoform of the NOX family in the kidney, is the major source of reactive oxygen species (ROS) that maintain normal cell physiology^7^. However, dysregulated NOX4 and oxidative stress might contribute to various diseases by causing mitochondrial injury, altered mitophagy, programmed cell death, and other mechanisms. For instance, NOX4 enhances ferroptosis in astrocytes by impairing mitochondrial metabolism in Alzheimer's disease^8^. Reestablishing the NOX4 redox balance via NOX4 blockade or mitochondria-specific ROS inhibitor treatment ameliorated the disturbance of mitophagy and attenuated susceptibility to acute exacerbation of chronic obstructive pulmonary disease^9^. Our previous study revealed that genetic or pharmacological inhibition of NOX4 effectively protects against sepsis-induced AKI by suppressing mitochondrial fission and apoptosis^10^. Silencing NOX4 also notably alleviated the levels of oxidative stress and ferroptosis in ischemia‒reperfusion injury (IRI)-induced AKI^11^.

The literature suggests that the NOX family plays a role in mediating the therapeutic effect of rIPC in some diseases. Knockdown of NOX1 in cultured cardiomyocytes abrogated the protective effect of IPC against hypoxia-induced apoptosis^12^. In hepatic IR injury, rIPC limits microcirculatory dysfunction, but this protection mainly affects NOX2^13^. Through RNA sequencing, we found that the genetic expression of NOX4 was substantially inhibited in rIPC-treated AKI mice, but whether rIPC protects AKI in a NOX4-dependent manner awaits further verification. Although AKI mice models revealed that rIPC effectively improved kidney dysfunction^14^-^16^, the renoprotection effect of early or late-phase rIPC remains to be verified. In this study, we hypothesized that rIPC protects against AKI by inhibiting NOX4-ROS signaling. By using different AKI models, including cisplatin-AKI, lipopolysaccharide (LPS)-AKI and IRI-AKI models, we aimed to validate the therapeutic efficacy of different rIPC strategies. Furthermore, we evaluated the NOX4-dependent renoprotective effect of rIPC by knocking out, silencing, or overexpressing NOX4 and investigated the potential underlying molecular mechanisms, which might help to quantify the biological effect of rIPC and optimize protocol implementation.

## Materials and Methods

### Reagents

Cisplatin (D8810) was acquired from SolarBio (Beijing, China). LPS (L8880) was purchased from SolarBio (Beijing, China). GKT137831 (S7171) was purchased from Selleck (Shanghai, China). Negative control (NC) siRNA (siNC) and NOX4 siRNA (siNOX4) were purchased from RiboBio (Guangzhou, China). Adenoviruses expressing NOX4 (Ad-NOX4) or not expressing NOX4 (Ad-Null) were purchased from Hanbio Tech (Shanghai, China).

### Animals

**Figure 1:**
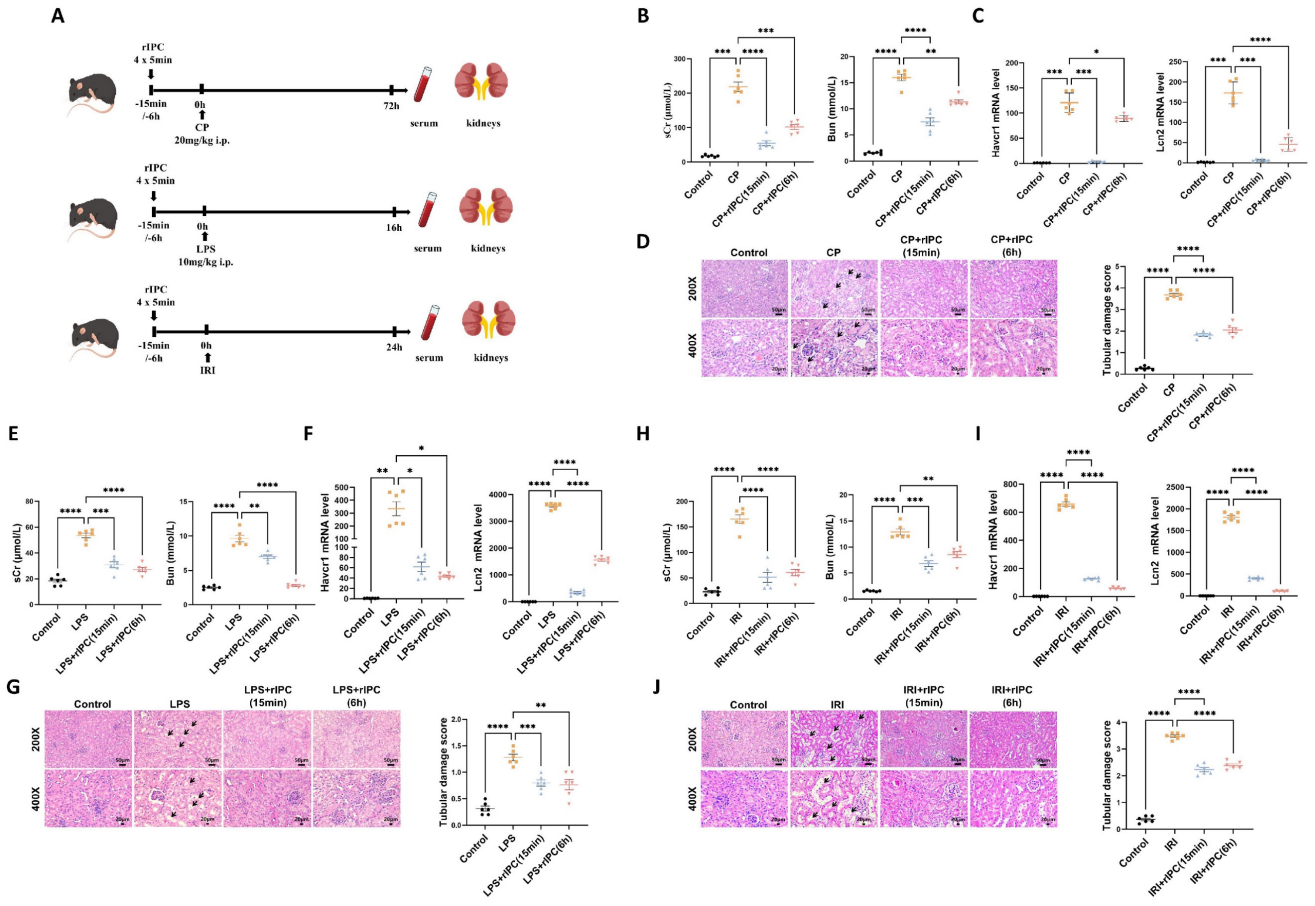
** rIPC protects renal function and renal tubule injury in mice with AKI.** (A) rIPC intervention in CP/LPS/IRI-induced AKI mouse models. (B) sCr and BUN levels in difference groups of CP-AKI mice. (C) Havcr1 and Lcn2 expression measured by RT-qPCR in CP-AKI mice kidney. (D) Representative image of hematoxylin and eosin (H&E) staining in CP-AKI mice kidney (200x, scale bar = 50μm; 400x, scale bar = 20 μm). (E) sCr and BUN levels in difference groups of LPS-AKI mice. (F) Havcr1 and Lcn2 expression measured by RT-qPCR in LPS-AKI mice kidney. (G) Representative image of hematoxylin and eosin (H&E) staining in LPS-AKI mice kidney (200x, scale bar = 50μm; 400x, scale bar = 20 μm). (H) sCr and BUN levels in difference groups of IRI-AKI mice. (I) Havcr1 and Lcn2 expression measured by RT-qPCR in IRI-AKI mice kidney. (J) Representative image of hematoxylin and eosin (H&E) staining in IRI-AKI mice kidney (200x, scale bar = 50μm; 400x, scale bar = 20 μm). Data are presented as mean ± SD, n = 6. Ripc: remote ischemic preconditioning, CP: cisplatin, LPS: lipopolysaccharides, IRI: ischemia/reperfusion injury, sCr: serum creatinine, BUN: blood urea nitrogen, Lcn2: (neutrophil gelatinase-associated lipocalin, NGAL) and Havcr1: (kidney injury molecule 1, KIM1). *p<0.05, **p<0.01, ***p<0.001, ****p<0.0001, #p<0.05, ##p<0.01, ###p<0.001, ####p<0.0001.

Six- to eight-week-old male C57BL/6J mice weighing 23-25g were procured from GemPharmatech (Chengdu, China). The Experimental Animal Ethics Committee at West China Hospital, Sichuan University approved the animal experiments (20220408008). Cisplatin, LPS, and IRI-induced AKI models were established (**Figure 1A**). A cisplatin-induced AKI mice model was created by injecting cisplatin (20 mg/kg) intraperitoneally. The details on the establishment of the LPS- and IRI-induced AKI mouse models are provided in the Supplementary Methods. For the GKT137831 treatment group, mice were intragastrically administered GKT137831 (60 mg/kg/d) for 6 consecutive days. Using a random number table method, a total of 126 mice were randomzied (24 for ciplatin-AKI model experiment, 24 for LPS-AKI model experiment, 24 for IRI-AKI model expriment, 18 for the GKT inhibition experiment, 18 NOX4^tecko^ mice and 18 NOX4^flox/flox^ mice) with 6 in each experimental group according to previous literature^10^, ^17^. Feeding and housing conditions were otherwise identical. No further eligibility criteria were set and there was no exclusion of animals in the analysis. The researchers were blinded for allocation during the outcome assessment and data analysis. The details of NOX4^tecko^ mice are provided in **Supplementary Methods, Figure S1-S2**.

### Cell culture and treatments

Mouse renal tubular epithelial cells (TCMK-1) were acquired from the American Type Culture Collection (Manassas, VA, USA) and cultured in DMEM supplemented with 10% fetal bovine serum (FBS) at 37°C in an environment of 95% air and 5% CO_2_. The TCMK-1 cells were exposed to cisplatin (2 μg/ml) for 24 hours. The optimal dosage of carbonyl cyanide 3-chlorophenylhydrazone (CCCP) was determined by a pre-experiment (**Figure S3**). The details of the siNOX4 and Ad-NOX4 transfections are provided in the Supplementary Methods.

### Remote ischemic preconditioning

The bilateral femoral arteries were clamped in sutures and subsequently subjected to 4 cycles of 5 minutes of ischemia followed by 5 minutes of reperfusion. In clinical practice, “early rIPC” usually implies a time interval between rIPC and AKI stimulus within 1 hour, while a “late rIPC” time interval could last up to hours and even days^18^. Accordingly, after a time interval of 15 minutes (early rIPC strategy)^16^ and 6 hours (late rIPC strategy) between rIPC and AKI challenge^15^, AKI was induced via cisplatin, LPS, or IRI. CCCP is an inhibitor of mitochondrial oxidative phosphorylation which is used to model the influence of mitochondrial uncoupling on ischemic-cell injury^19^. Brief treatment with CCCP has been established as an *in vitro* model of rIPC by inducing “chemical” ischemic-preconditioning^20^. In this study, TCMK-1 cells were pretreated with CCCP (2 μg/ml, 10 mM) for 30 min to mimic rIPC^20^.

### RNA-sequencing analysis

Frozen kidney samples from the control, cisplatin-induced AKI model and rIPC groups (n=3 per group) were randomly selected for sequencing. Total RNA was extracted using Trizol reagent (thermofisher, 15596018). After total RNA was analyzed for purity, quality, and integrity, library construction and sequencing were performed by LC-BIO Bio-Tech Ltd. (Hangzhou, China). Subsequently, the 2 x 150bp paired-end sequencing were performed on Illumina Novaseq™ 6000 (LC-Bio Technology CO., Ltd., Hangzhou, China). A fold change greater than 2 and a p-value less than 0.05 were considered differentially expressed genes.

### Renal function evaluation and histologic examination

Blood samples were centrifuged at 3000 rpm for 30 minutes to obtain serum. The levels of serum creatinine (sCr) and blood urea nitrogen (BUN) were detected via an automatic biochemical analyzer (Mindray BS-240, Shenzhen, China). The kidney tissue was fixed in a 10% formaldehyde solution (50-00-0; Chron Chemicals, Chengdu, China) and subjected to histological analysis via hematoxylin and eosin (H&E) staining. The Supplementary Methods provided details on the H&E staining and evaluation procedures.

### Immunohistochemistry

Kidney tissues were embedded in OCT compound and frozen at a temperature of -80 °C. Kidney tissue sections (4 μm) were subjected to standard procedures, including dewaxing, dehydration, and antigen retrieval. Subsequently, the sections were blocked with goat serum (1:200) for 30 min at 37°C, and the primary antibody against NOX4 (1:200) was added and incubated at 4°C for 24 hours. The sections were cleaned in PBS and stained with the VECTASTAIN ABC Kit (Vector, Burlingame, CA, USA). Finally, images of the sections were obtained using an AxioCamHRc digital camera (Carl Zeiss, Jena, Germany) with ZEN 2012 microscopy software at 200x magnification. The intensity of the immunohistochemical staining was analyzed using ImageJ software (version 1.51; Wayne Rasband, NIH, USA).

### Immunofluorescence staining

Kidney tissue sections (4 μm) were subjected to standard procedures, including dewaxing, dehydration, and antigen retrieval. These sections were subsequently blocked with goat serum (1:200) for 1 hour at 37°C. The primary antibodies against NOX4 (1:200) and GPX4 (1:200) were incubated with the sections at 4°C for 24 hours. The sections were washed in PBS. Fluorescein-labeled Lotus quadrangular lectin was used to locate the proximal tubules, and DAPI was used to locate the cell nuclei. Finally, the images of the sections were observed using an AxioCamHRc digital camera (Carl Zeiss, Jena, Germany) with ZEN 2012 microscopy software at 200x magnification. The intensity of immunofluorescence was analyzed using ImageJ software.

### Reactive oxygen species detection

The level of ROS in the kidney tissue was detected by using the oxidative fluorescent dye dihydroethidium (DHE) (Sigma‒Aldrich, St. Louis, MO, USA). The images were obtained with fluorescence microscopy (Nikon, Tokyo, Japan). The ROS production levels in TCMK-1 cells was measured with an ROS assay kit (S0033M; Beyotime Biotechnology, Shanghai, China). The intensity of ROS was analyzed using ImageJ software.

### Transmission electron microscopy

Kidney tissue was fixed with 3% glutaraldehyde for 2 hours at 4°C. Subsequently, the tissue was subjected to standard procedures, including dehydration, embedding, ultrathin sectioning, and staining. Finally, the tissue was visualized with a transmission electron microscope (JEM-1400-FLASH, Tokyo, Japan).

### Measurement of GSH levels in kidney tissues

Glutathione (GSH) levels in kidney tissues were measured according to the kit instructions (S0053, Beyotime Biotechnology, Shanghai, China). The contents of GSH were detected at 412 nm.

### Measurement of MDA levels in kidney tissue

MDA activity was detected in kidney tissues according to standard protocols (S0131M, Beyotime Biotechnology, Shanghai, China). The MDA contents were detected at 532 nm. The MDA concentration was normalized to the total protein concentration.

### Western blot

Two samples per group were randomly selected for western blotting. The workflow for western blot analysis was performed as described in a previous study^10^. Subsequently, protein densitometry analysis was performed with ImageJ software, with GAPDH serving as an internal standard protein. The primary antibodies used are listed in **Table S1**.

### Quantitative real-time PCR

Three samples per group were randomly selected for quantitative real-time PCR (RT‒qPCR). RT-qPCR was performed as described in a previous study^10^. The primers used for the genes are listed in **Table S2**. The mRNA expression levels of related genes were analyzed by CFX Manager™ Software (Bio-Rad, Hercules, CA, USA) with GAPDH serving as an internal standard gene.

### Statistical analysis

The experiments were repeated three times, and the data are presented as means ± standard deviations. The Mann‒Whitney U test or two-tailed Student's t test was used to analyze the differences between two groups. The differences between multiple groups were analyzed by using ANOVA, followed by Tukey's multiple comparisons test. GraphPad Prism 9.3.1 (GraphPad Software, San Diego, CA, USA) was used for statistical analysis. A p-value of less than 0.05 was considered statistically significant.

## Results

### rIPC protects renal function and renal tubule injury in AKI

The protective effects of rIPC on AKI were validated using cisplatin, LPS, and IRI-AKI mouse models. Biochemical analysis demonstrated that rIPC, whether administered 15 min or 6 h before AKI insult, significantly reduced the levels of both sCr and BUN in cisplatin-AKI mice (**Figure 1B**). The gene expression of KIM-1 (Havcr1) and NGAL (Lcn2) was also notably decreased in cisplatin-AKI mouse kidneys treated with rIPC, as shown by RT-qPCR (**Figure 1C**). H&E staining and histological analysis revealed that rIPC treatment ameliorated tubular dilatation, brush border loss, and tubular epithelial vacuolation and significantly decreased the tubular damage score (**Figure 1D**). Additionally, the therapeutic effect of rIPC was verified in vitro (**Figure 3A**). As illustrated in **Figure 3B**, the tubular injury markers KIM-1 (Havcr1) and NGAL (Lcn2) were downregulated after CCCP treatment. Consistent results were observed in the LPS- and IRI-AKI mouse models (**Figure 1E-J**). Taken together, these findings support the notion that rIPC protects renal function and attenuates kidney tubule injury in AKI.

### rIPC ameliorates inflammation and oxidative stress in AKI

**Figure 2:**
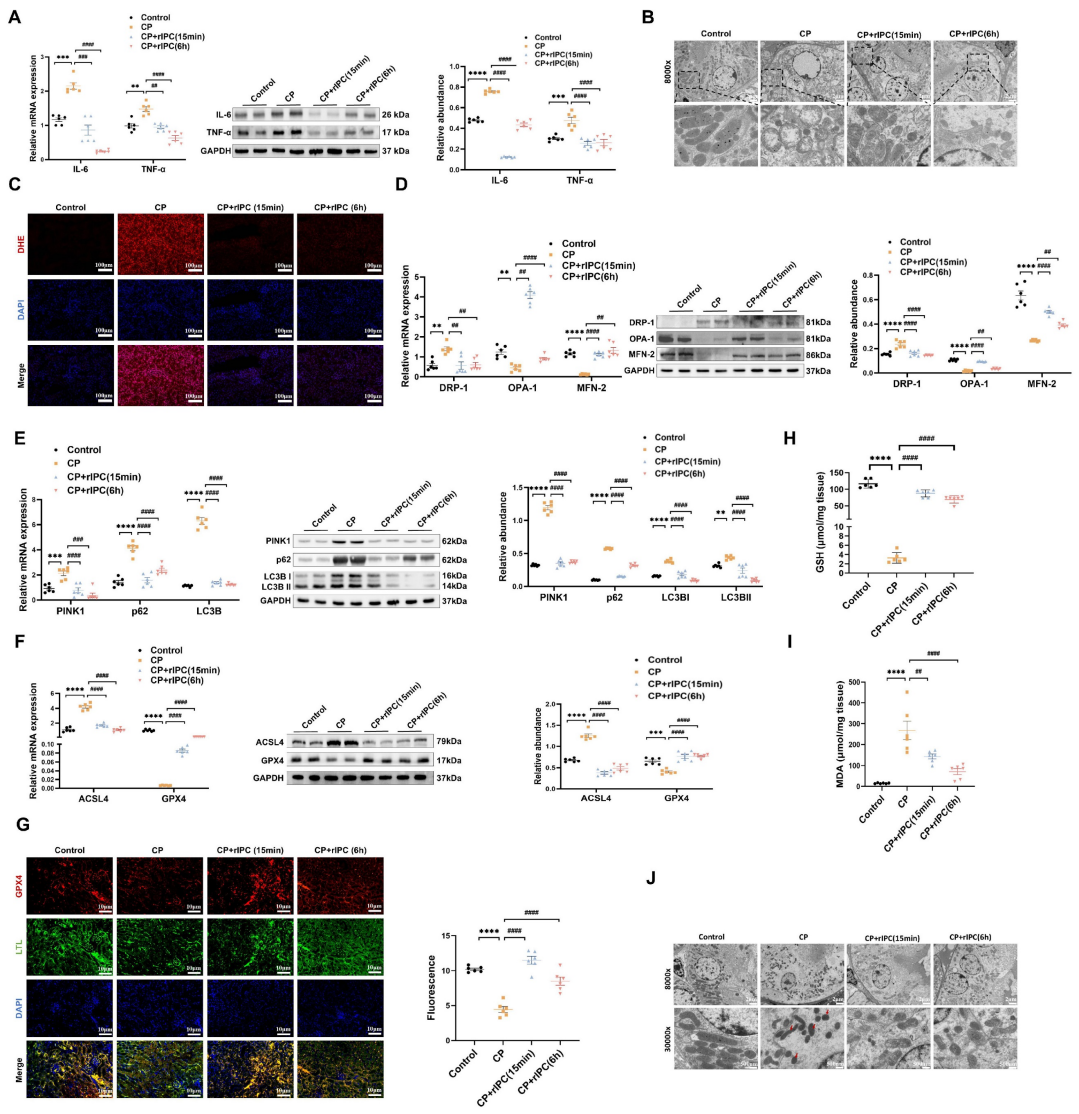
** rIPC attenuates inflammation, oxidative stress, mitochondrial malfunction and ferroptosis in AKI mice.** (A) IL-6 and TNF-α expression by RT-qPCR and western blot in CP-AKI mouse kidney. (B) The morphology of mitochondria under transmission electron microscope of CP-AKI mouse kidney (8000x, scale bar = 2μm). (C) ROS assessed by DHE staining in CP-AKI mouse kidney (200x, scale bar = 100 μm). (D) Mitochondrial dynamic regulatory molecules (DRP-1, OPA-1 and MFN-2) measured by RT-qPCR, western blot and quantified by densitometry in CP-AKI mouse kidney. (E) Mitophagy level (PINK1, p62/SQSTM1 and LC3B) measured by RT-qPCR, western blot and quantified by densitometry in CP-AKI mouse kidney. (F) Ferroptosis-regulatory molecules (ACSL4 and GPX4) measured by RT-qPCR, western bolt and quantified by densitometry in CP-AKI mouse kidney. (G) Representative image of immunofluorescence staining of GPX4 in CP-AKI mouse kidney (200x, scale bar = 10 μm). (H) The levels of GSH in CP-AKI mouse kidney tissue. (I) The levels of MDA in CP-AKI mouse kidney tissue. (J) The morphological characteristic of ferroptosis under transmission electron microscope of CP-AKI mouse kidney (8000x, scale bar = 2μm, 30000x, scale bar = 500nm). Data are presented as mean ± SD, n = 6. rIPC: remote ischemic preconditioning, CP: cisplatin, ROS: reactive oxygen species, GSH: glutathione. *p<0.05, **p<0.01, ***p<0.001, ****p<0.0001, #p<0.05, ##p<0.01, ###p<0.001, ####p<0.0001.

**Figure 3:**
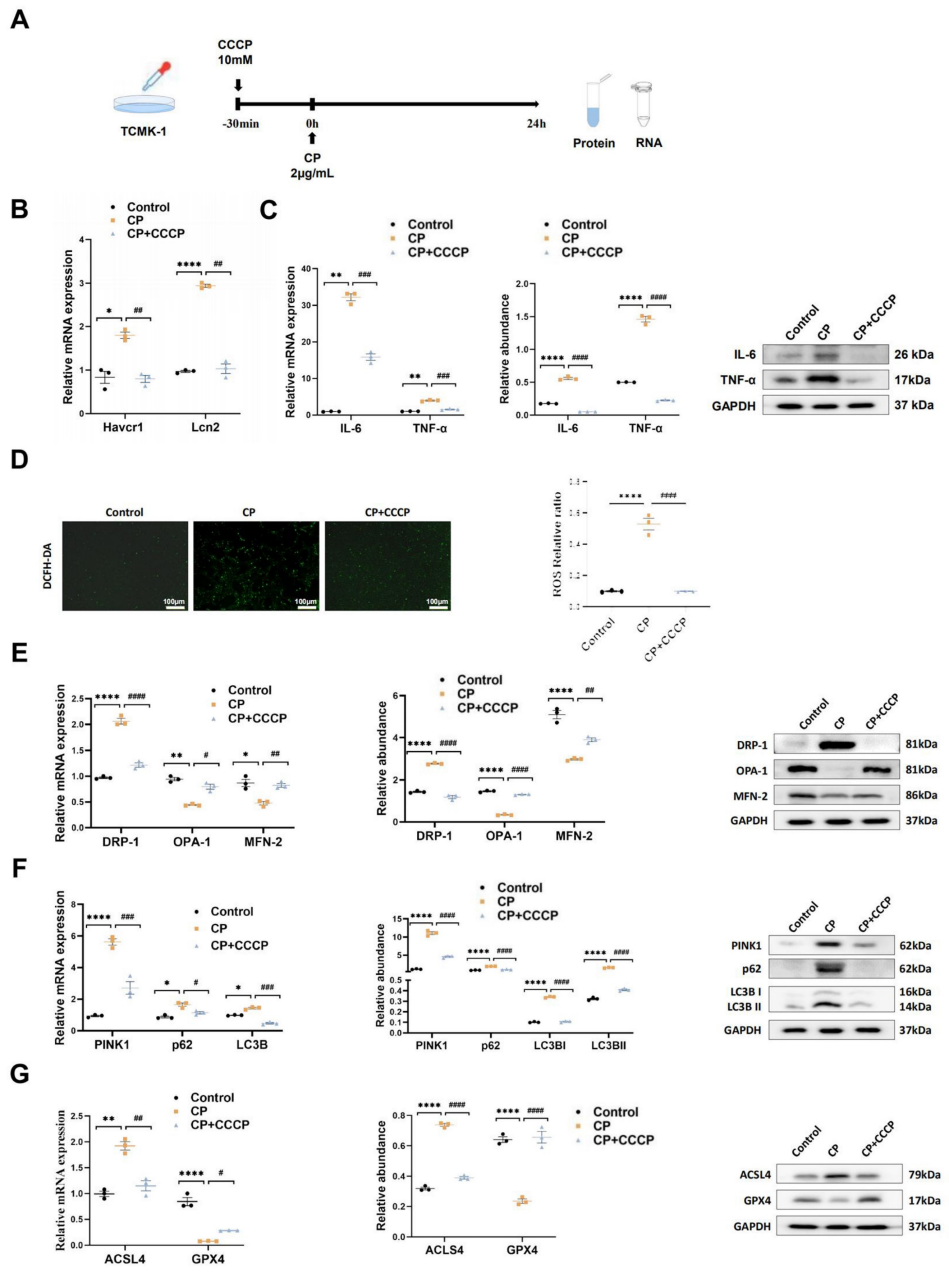
** rIPC attenuates inflammation, oxidative stress, mitochondrial malfunction and ferroptosis in TCMK-1 cells.** (A) rIPC intervention in cisplatin stimulated TCMK-1 cells. (B) Havcr1 and Lcn2 expression measured by RT-qPCR. (C) IL-6 and TNF-α expression analyzed by RT-qPCR, western blot and quantified by densitometry. (D) The ROS production in TCMK-1 cells was assessed by DCFH-DA staining (100x, scale bar = 100 μm). (E) Mitochondrial dynamic regulatory molecules (DRP-1, OPA-1 and MFN-2) assessed by RT-qPCR, western blot and quantified by densitometry. (F) Mitophagy level (PINK1, p62/SQSTM1 and LC3B) measured by RT-qPCR, western blot and quantified by densitometry in TCMK-1 cells. (G) Ferroptosis-regulatory molecules (ACSL4 and GPX4) measured by RT-qPCR, western blot and quantified by densitometry. Data are presented as mean ± SD, n = 3. CP: cisplatin, CCCP: carbonyl cyanide 3-chlorophenylhydrazone, ROS: reactive oxygen species, Lcn2: (neutrophil gelatinase-associated lipocalin, NGAL) and Havcr1: (kidney injury molecule 1, KIM1). *p<0.05, **p<0.01, ***p<0.001, ****p<0.0001, #p<0.05, ##p<0.01, ###p<0.001, ####p<0.0001.

We evaluated the expression of inflammatory markers. In cisplatin-AKI, rIPC significantly reduced the TNF-a and IL-6 mRNA and protein levels in both mouse kidneys and TCMK-1 cells (**Figure 2A, Figure 3C**). A similar trend in inflammation was observed in the LPS- and IRI-AKI models (**Figure S4A, Figure S5A**). With regard to oxidative stress, dihydroethidium (DHE) staining showed that the level of oxidative stress in cisplatin-AKI mouse kidney tissues was attenuated by rIPC *in vivo* (**Figure 2C**). Pretreatment with CCCP significantly alleviated the increase in ROS fluorescence in cisplatin-challenged TCMK-1 cells *in vitro* (**Figure 3D**). Thus, rIPC attenuates inflammation and oxidative stress in AKI.

### rIPC rebalances mitochondrial dynamics and mitophagy in AKI

Mitochondria are the center of energy metabolism and are increasingly recognized as playing a key role in the physiopathology of AKI^21^. We further investigated the impact of rIPC on mitochondrial dynamics in AKI. RT-qPCR and western blotting showed that during AKI, DRP-1, which is responsible for mitochondrial fission, was significantly upregulated, while MFN-2 and OPA-1, which mediate mitochondrial fusion, were downregulated. Both early and late rIPC treatment successfully reversed the dynamic imbalance toward fission and restored mitochondrial homoeostasis in cisplatin-treated mice and TCMK-1 cells (**Figure 2D, Figure 3E**). The morphology of the mitochondria was examined using a transmission electron microscope (TEM). We observed that AKI mouse tubular epithelial cells exhibited ultrastructural changes in mitochondrial morphology, including swelling, cristae loss, fragmentation, and vacuoles in the mitochondrial matrix. These changes were notably alleviated by rIPC treatment (**Figure 2B**). Similarly, the protective effects of rIPC on mitochondrial dynamics and morphology were also recorded in LPS- and IRI-AKI model mice (**Figure S4B-S4C, Figure S5B-S5C**). As a stress response mechanism, mitophagy is a specific form of autophagy that can be induced in various pathological processes, such as inflammation and oxidative stress. Both *in vivo* and *in vitro* experiments revealed that during cisplatin-AKI, mitophagy mediators such as p62, LC3B, and PINK1 were overexpressed, indicating a state of aberrant autophagic flux characterized by increased mitophagy induction and altered autophagosome fusion. Treatment with rIPC alleviated the tubular epithelial mitophagy disturbance (**Figure 2E, Figure 3F**). Observations of LPS- and IRI-AKI mice revealed similar findings for mitophagy (**Figure S4D, Figure S5D**). Our results indicated that rIPC plays a protective role in restoring mitochondrial dynamics and mitophagy during AKI.

### rIPC reduces lipid peroxidation and ferroptosis in AKI

Ferroptosis, a newly characterized form of cell death characterized by iron-dependent lipid peroxidation, has been reported to play an important role in the pathogenesis of AKI. As shown by RT-qPCR and western blot analysis, cisplatin substantially increased the expression of the key ferroptosis mediator ACSL4 and decreased GPX4 expression, thus promoting ferroptosis, while rIPC reversed the changes in ACSL4 and GPX4 levels in AKI mouse kidneys and TCMK-1 cells (**Figure 2F, Figure 3G**). Consistently, rIPC also reversed the decreased GSH levels in cisplatin-induced AKI mouse kidneys (**Figure 2H**). Immunofluorescence image analysis suggested that rIPC significantly enhanced the intensity of GPX4 in kidney sections from cisplatin-AKI mice (**Figure 2G**). In addition, the morphological characteristic of ferroptosis was detected using TEM. We observed obviously shrunken mitochondria, increased membrane density and diminished crista in tubular epithelial cells of kidneys after cisplatin treatment, which represented a characteristic morphologic feature of ferroptosis. These changes were notably alleviated by rIPC treatment (**Figure 2J**). These findings were further validated in LPS- and IRI-AKI (**Figure S4E-S4H; Figure S5E-S5H**), confirming that rIPC prevents ferroptosis in AKI. In addition, cisplatin increased the level of the lipid peroxidation product MDA in mouse kidneys, while rIPC effectively decreased the MDA concentration (**Figure 2I**). Similarly, rIPC also effectively decreased the MDA concentration in mice with LPS- and IRI-induced AKI (**Figure S4I, Figure S5I**). These results collectively demonstrated that rIPC reduces lipid peroxidation and tubular epithelial ferroptosis during AKI.

### rIPC reverses the upregulation of NOX4

**Figure 4:**
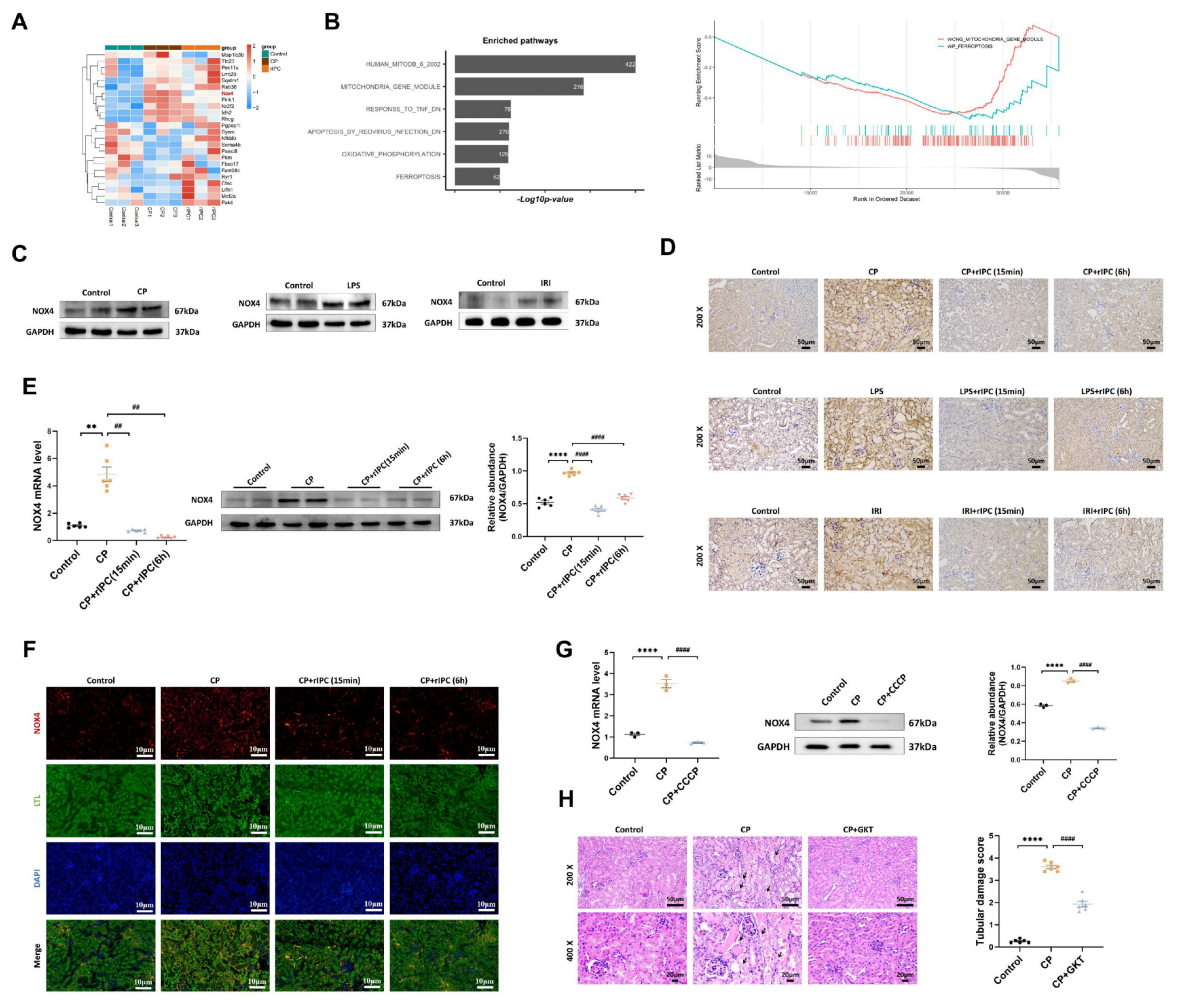
** rIPC reverses the upregulation of NOX4 in AKI.** (A) Representative heatmap of differentially expressed genes in the kidneys of CP-AKI mice (n=3). (B) Comparable analysis between cisplatin and cisplatin + rIPC group using Gene Set Enrichment Analysis (GSEA). (C) NOX4 expression assessed by Western blot in cisplatin, LPS and IRI-induced AKI. (D) Representative image of immunochemistry staining of NOX4 in kidney tissue sections (200x, scale bar = 50μm). (E) rIPC reverses the upregulation of NOX4 assessed by RT-qPCR, western blot and quantified by densitometry in mice kidney (n=6). (F) Representative image of immunofluorescence staining of NOX4 in CP-AKI mouse kidney (200x, scale bar = 10μm). (G) rIPC reverses the upregulation of NOX4 assessed by RT-qPCR, western blot and quantified by densitometry in TCMK-1 (n=3). (H) NOX4 inhibitor GKT137831 improved the pathological injury and tubular damage score in CP-induced AKI mice under hematoxylin and eosin (H&E) staining (200x, scale bar = 50μm; 400x, scale bar = 20 μm). Data are presented as mean ± SD, n = 6. rIPC: remote ischemic preconditioning, CP: cisplatin, CCCP: carbonyl cyanide 3-chlorophenylhydrazone, LPS: lipopolysaccharides, IRI: ischemia/reperfusion injury. *p<0.05, **p<0.01, ***p<0.001, ****p<0.0001, #p<0.05, ##p<0.01, ###p<0.001, ####p<0.0001.

RNA-seq analysis revealed significant genetic upregulation of NOX4 in cisplatin-AKI mice, while in rIPC-treated AKI mice, NOX4 expression decreased to a level comparable to that in the control group (**Figure 4A**), which suggested that NOX4 might be involved in the renoprotective effect of rIPC. Gene Set Enrichment Analysis (GSEA) revealed that mitochondria and ferroptosis were significantly enriched pathways in AKI and were markedly downregulated after rIPC treatment (**Figure 4B**). By analyzing public single-cell RNA sequencing data from The Kidney Precision Medicine Project (KPMP), NOX4 was found to be expressed abundantly in the proximal tubules. Furthermore, the expression of NOX4 in proximal tubule epithelial cells was evidently upregulated in AKI patients (**Figure S6**). In this study, we confirmed that NOX4 was substantially upregulated in cisplatin-, LPS- and IRI-induced AKI (**Figure 4C, Figure S7A-S7B**). Furthermore, we employed RT-qPCR, western blotting, immunohistochemistry, and immunofluorescence to study the influence of rIPC on NOX4 expression in cisplatin-AKI mice and cisplatin-treated TCMK-1 cells. The upregulation of NOX4 was mitigated by rIPC *in vivo* and *in vitro* (**Figure 4D-4G**). Together with the results of corresponding experiments performed on LPS- and IRI-AKI models (**Figure S7C-S7F**), these results consistently showed that rIPC reverses the upregulation of NOX4 at the genetic and protein levels. GKT137831, an inhibitor of NOX4, was orally administered to AKI mice. The levels of sCr and BUN in plasma and KIM-1 and NGAL in the kidney were significantly reduced after GKT137831 treatment (**Figure S7G**), as was the pathological injury and tubular damage score (**Figure 4H**). Therefore, rIPC reverses the upregulation of NOX4, while pharmacological inhibition of NOX4 with GKT137831 achieved renal protection similar to that of rIPC. NOX4 might play a mediating role in the effect of rIPC in treating AKI.

### rIPC attenuates mitochondrial malfunction and ferroptosis by inhibiting NOX4-ROS signaling in AKI

To elucidate the mechanism underlying the protective effects of rIPC in AKI, we analyzed the therapeutic efficacy of rIPC in cisplatin-induced AKI models with NOX4^tecko^ mice and TCMK-1 cells in which the NOX4 gene was silenced (siNOX4) or overexpressed by adenoviruses (Ad-NOX4). These models allowed us to further validate whether rIPC shields against AKI by inhibiting NOX4.

### 1. The protective effects of rIPC on TCMK-1 cells are mitigated by NOX4 overexpression

**Figure 5:**
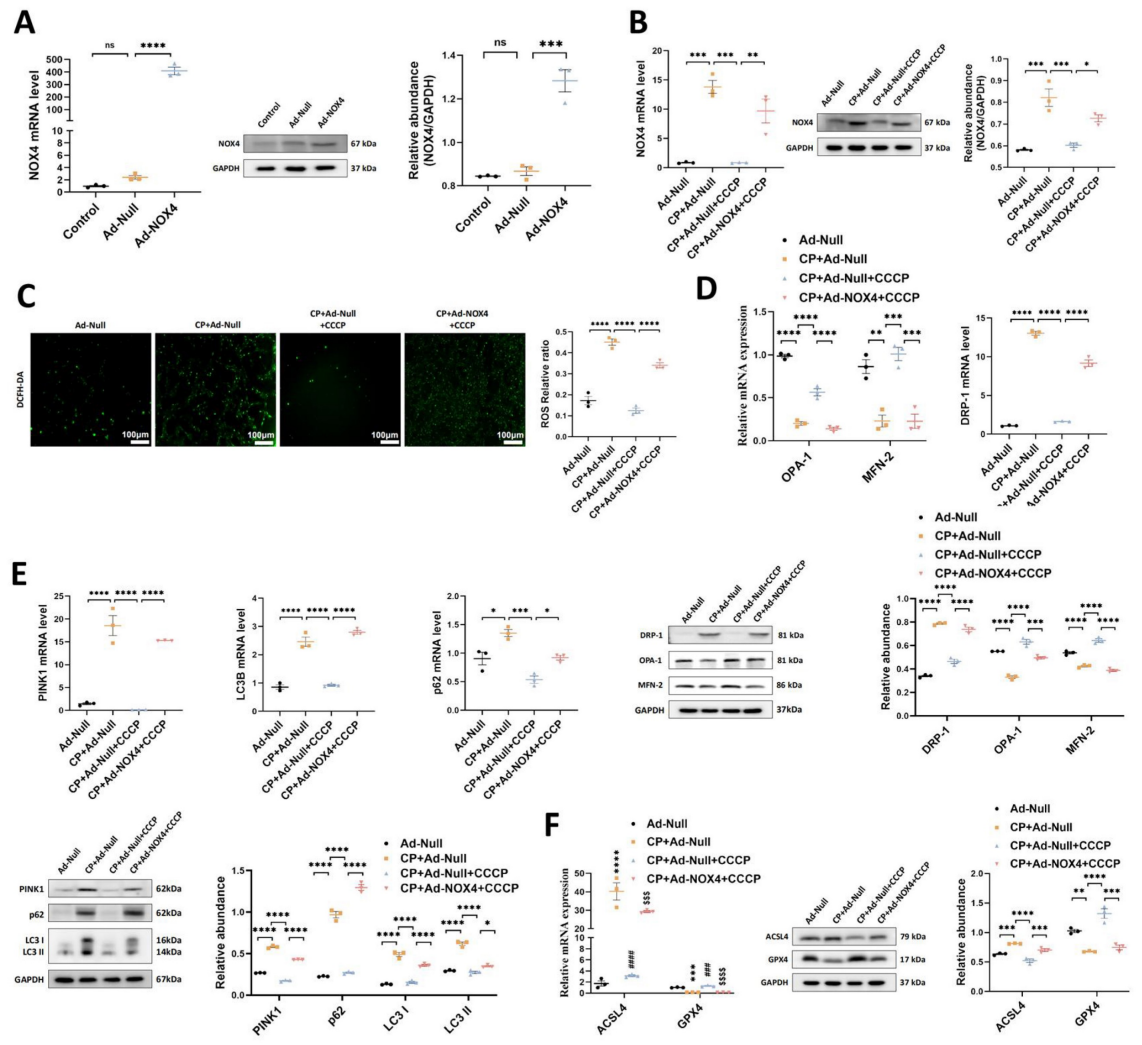
** The protective effects of rIPC on TCMK-1 cells are mitigated by NOX4 overexpression.** (A) TCMK-1 cells were transfected with negative control (NC) Ad-RNA or Ad-NOX4 for 24 h and then treated with 2 μg/ml cisplatin for 24 h. The overexpression efficiency of NOX4 in TCMK-1 cells was evaluated by RT-qPCR analysis, western blot analysis and quantified by densitometry. (B) NOX4 expression evaluated by RT-qPCR analysis, western blot analysis and quantified by densitometry. (C) The ROS production in TCMK-1 cells was assessed by DCFH-DA staining (100x, scale bar = 100 μm). (D) Mitochondrial dynamic regulatory molecules (DRP-1, OPA-1 and MFN-2) evaluated by RT-qPCR analysis and western blot analysis. (E) Mitophagy level (PINK1, p62/SQSTM1 and LC3B) assessed by RT-qPCR analysis and western blot analysis. (F) Ferroptosis-regulatory molecules (ACSL4 and GPX4) assessed by RT-qPCR analysis, western blot analysis and quantified by densitometry. Data are presented as mean ± SD, n = 3. CCCP: carbonyl cyanide 3-chlorophenylhydrazone, CP: cisplatin, ROS: reactive oxygen species. *p<0.05, **p<0.01, ***p<0.001, ****p<0.0001, #p<0.05, ##p<0.01, ###p<0.001, ####p<0.0001, ns: no significant.

To confirm the mediating role of NOX4 in rIPC, we repeated *in vitro* experiments using TCMK-1 cells transfected with Ad-NOX4. The cells were stimulated with different doses of Ad-NOX4 to determine the optimal dose and timing (**Figure S8**), and we adopted a strategy in which TCMK-1 cells were stimulated with Ad-NOX4 at an MOI of 1000 for 24 hours. As illustrated in **Figure 5A**, the expression of NOX4 was significantly upregulated in the TCMK-1 cells transfected with Ad-NOX4. The protein and mRNA levels of NOX4 both increased after cisplatin stimulation. CCCP decreased NOX4 expression, which was again enhanced by Ad-NOX4 transfection (**Figure 5B**). CCCP effectively downregulated ROS production, restored mitochondrial dynamics and mitophagy, and inhibited ferroptosis in cisplatin-stimulated TCMK-1 cells. However, the ability of CCCP to protect against mitochondrial malfunction and ferroptosis was abrogated in cells transfected with Ad-NOX4 (**Figure 5C-5F**).

### 2. The protective effects of rIPC on TCMK-1 cells are enhanced by NOX4 silencing

**Figure 6:**
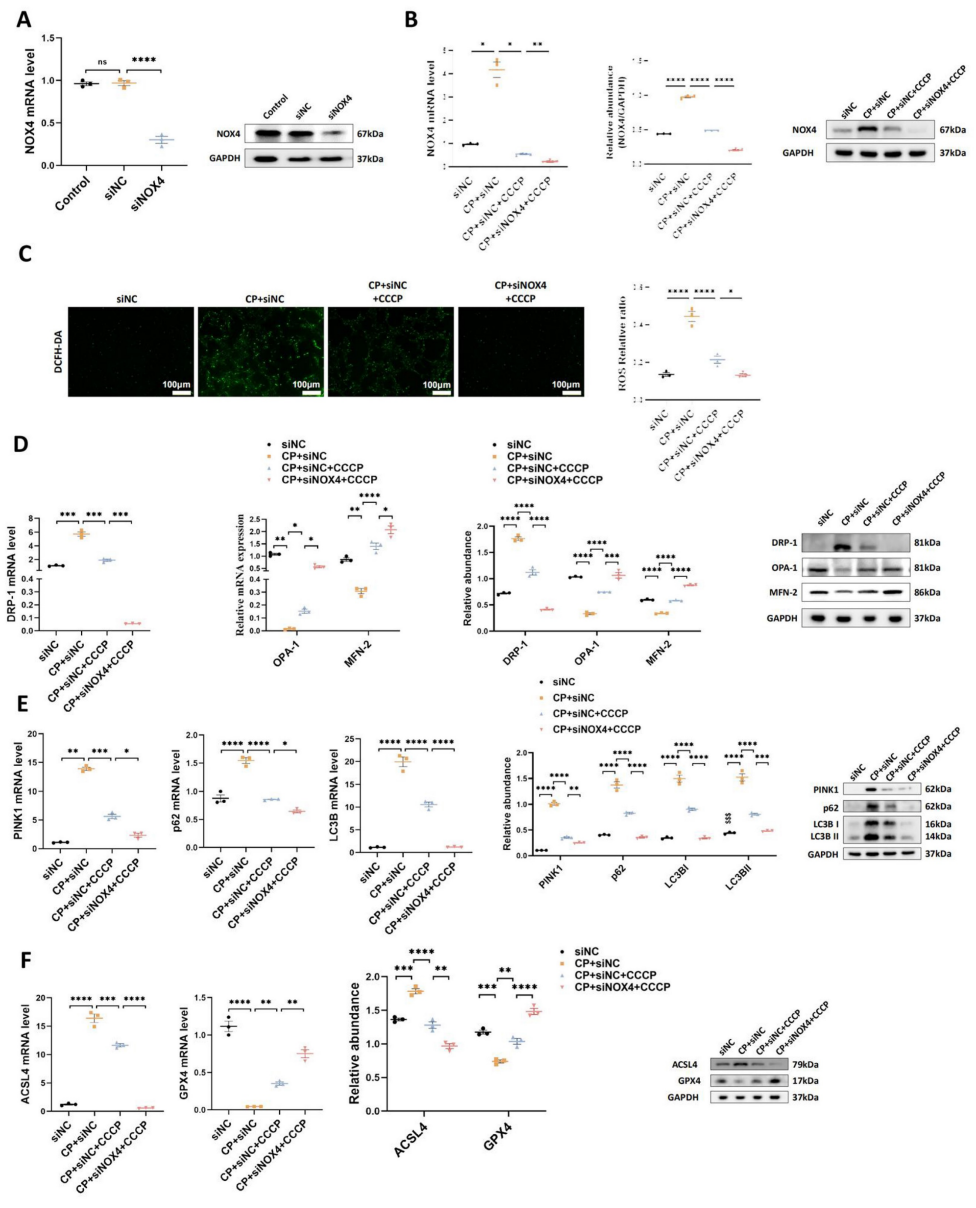
** The protective effects of rIPC on TCMK-1 cells are enhanced by NOX4 silencing.** (A) TCMK-1 cells were transfected with negative control (NC) siRNA or NOX4 siRNA for 6 h and then treated with 2 μg/ml cisplatin for 24 h. The knockdown efficiency of NOX4 siRNA in TCMK-1 cells was evaluated by RT-qPCR analysis and western blot analysis. (B) NOX4 expression evaluated by RT-qPCR analysis, western blot analysis and quantified by densitometry. (C) The ROS production in TCMK-1 cells was assessed by DCFH-DA staining (100x, scale bar = 100 μm). (D) Mitochondrial dynamic regulatory molecules (DRP-1, OPA-1 and MFN-2) evaluated by RT-qPCR analysis, western blot analysis and quantified by densitometry. (E) Mitophagy level (PINK1, p62/SQSTM1 and LC3B) measured by RT-qPCR analysis, western blot analysis and quantified by densitometry. (F) Ferroptosis-regulatory molecules (ACSL4 and GPX4) assessed by RT-qPCR analysis, western blot analysis and quantified by densitometry. Data are presented as mean ± SD, n = 3. CCCP: carbonyl cyanide 3-chlorophenylhydrazone, CP: cisplatin, ROS: reactive oxygen species. *p<0.05, **p<0.01, ***p<0.001, ****p<0.0001.

To validate the synergistic effect of NOX4 inhibition and rIPC, we conducted *in vitro* experiments using TCMK-1 cells transfected with siNOX4. As illustrated in **Figure 6A-6B**, similar to CCCP, silencing the NOX4 gene successfully decreased the mRNA and protein expression of NOX4. CCCP ameliorated oxidative stress, mitochondrial malfunction and ferroptosis in wild-type TCMK-1 cells stimulated with cisplatin, which showed additional improvement in siNOX4-transfected TCMK-1 cells (**Figure 6C-6F**).

### 3. The protective effects of rIPC on AKI mice are enhanced by NOX4 knockout

**Figure 7:**
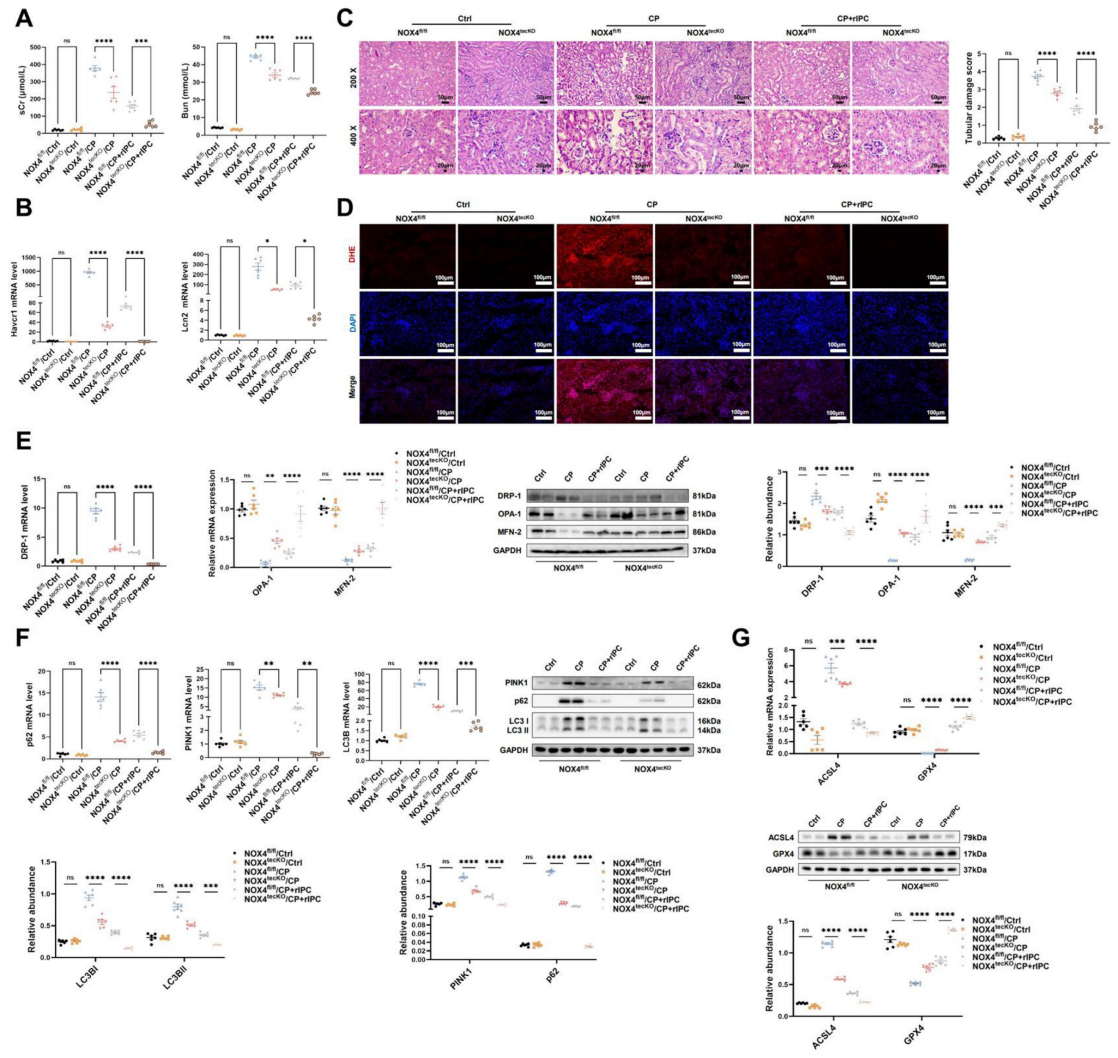
** The protective effects of rIPC on AKI mice are enhanced by NOX4 knockout.** (A) sCr and BUN levels in difference groups of CP-AKI mice. (B) Havcr1 and Lcn2 expression measured by RT-qPCR in CP-AKI mouse kidney. (C) Representative image of hematoxylin and eosin (H&E) staining in CP-AKI mouse kidney (200x, scale bar = 50μm; 400x, scale bar = 20 μm). (D) ROS assessed by DHE staining in CP-AKI mouse kidney (200x, scale bar = 100 μm). (E) Mitochondrial dynamic regulatory molecules (DRP-1, OPA-1 and MFN-2) analyzed by RT-qPCR, western blot and quantified by densitometry in CP-AKI mouse kidney. (F) Mitophagy level (PINK1, p62/SQSTM1 and LC3B) analyzed by RT-qPCR, western blot and quantified by densitometry in CP-AKI mouse kidney. (G) Ferroptosis-regulatory molecules (ACSL4 and GPX4) assessed by RT-qPCR, western bolt and quantified by densitometry in CP-AKI mouse kidney. Data are presented as mean ± SD, n = 6. rIPC: remote ischemic preconditioning, CP: cisplatin, Lcn2: (neutrophil gelatinase-associated lipocalin, NGAL) and Havcr1: (kidney injury molecule 1, KIM1), ROS: reactive oxygen species. *p<0.05, **p<0.01, ***p<0.001, ****p<0.0001, #p<0.05, ##p<0.01, ###p<0.001, ####p<0.0001, ns: no significant.

**Figure 8:**
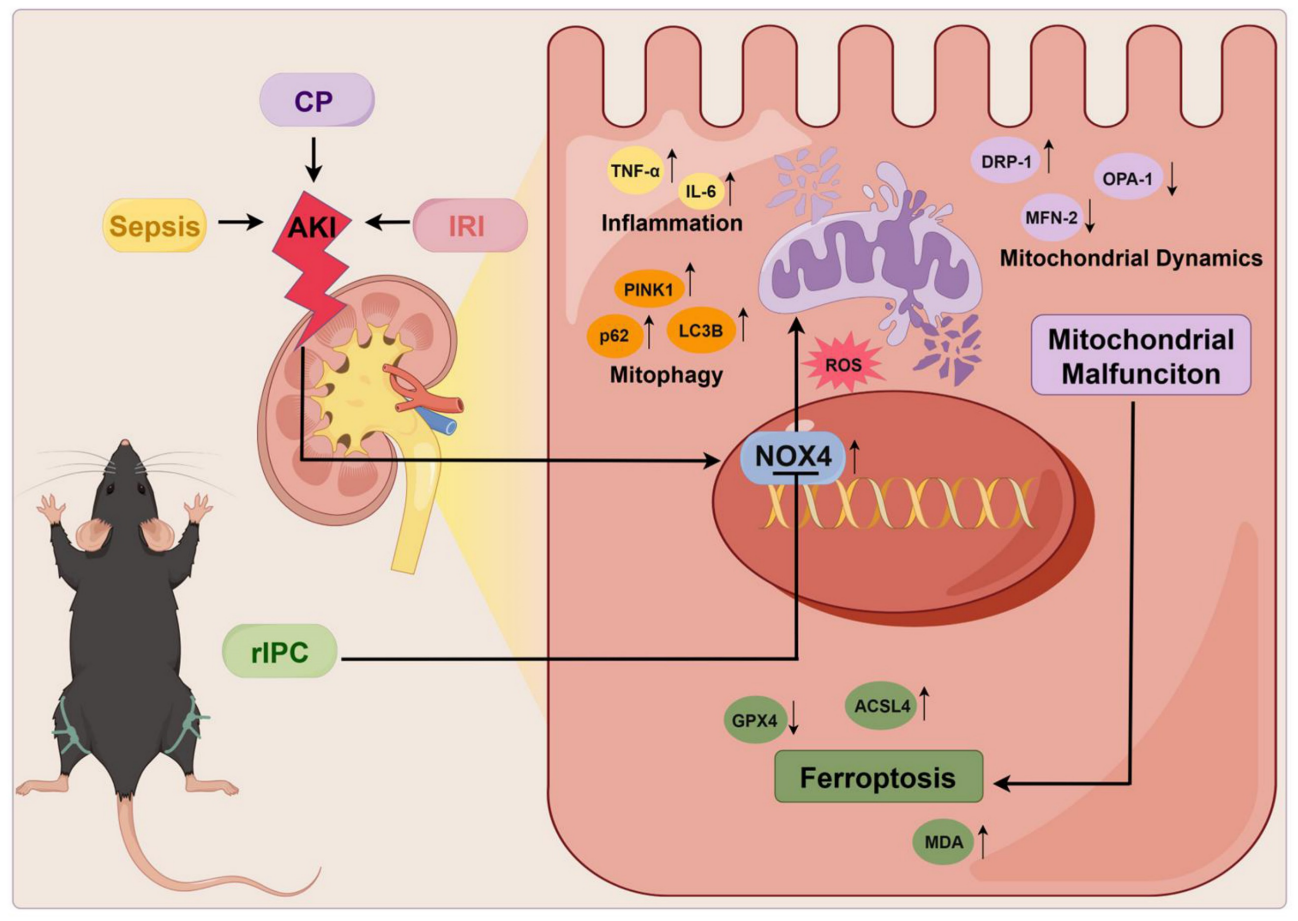
** Mechanism of remote ischemic preconditioning in the protection against acute kidney injury.** rIPC: remote ischemic preconditioning, CP: cisplatin, IRI: ischemia/reperfusion injury, AKI: acute kidney injury.

In the cisplatin-induced AKI model, rIPC effectively reduced the levels of sCr and BUN in wild-type mice. These reductions were more prominent in NOX4^tecko^ mice treated with rIPC (**Figure 7A**). Additionally, we investigated pathological injury and KIM-1 and NGAL levels, which exhibited similar trends (**Figure 7B-7C**). These findings suggested that inhibiting NOX4 enhanced the protective effects of rIPC on renal function and tubular injury, suggesting the synergistic effect of NOX4 inhibition and rIPC in treating AKI. Furthermore, in wild-type AKI mice, rIPC effectively decreased ROS production, restored the imbalance in mitochondrial dynamics and mitophagy, and ameliorated tubular epithelial ferroptosis. Notably, the protective effects of rIPC were further enhanced in NOX4^tecko^ AKI mice (**Figure 7D-7G**). Therefore, overexpressing NOX4 abrogated the therapeutic efficacy of rIPC, while inhibiting NOX4 enhanced its protective effect. rIPC might attenuate mitochondrial dysfunction, reduces ferroptosis and protects against AKI in a NOX4-dependent manner (**Figure 8**).

## Discussion

AKI is associated with a significant incidence of morbidity and mortality, yet effective treatments are unavailable. Therefore, the exploration of novel therapeutic alternatives for AKI is urgently warranted. Ischemic preconditioning (IPC) was first described by Murry et al. in 1986; their findings suggested that multiple brief ischemic episodes might protect the heart from subsequent sustained ischemic insult^22^. Interestingly, the protective effect of IPC does not necessarily originate from the organ to be protected itself. In other words, IPC can be performed in distant tissues and activate messengers to activate protective signaling pathways in the target organ; this process is also known as remote IPC (rIPC)^23^. For decades, researchers have attempted to use rIPC to protect the functions of organs, including the heart, brain, and kidney^24^-^27^. However, no consensus has been reached on its definitive therapeutic efficacy. A meta-analysis of randomized controlled trials showed that rIPC did not reduce overall morbidity or mortality in patients undergoing cardiac surgery^28^, but a reduction in the incidence of cardiac surgery-associated AKI was observed^5^. Given the complexity of AKI etiology, the expandability of findings on surgery-associated AKI to other forms of AKI remains underinvestigated. Wang *et al.* reported that remote liver ischemic preconditioning has a renoprotective effect on IRI-AKI injury, which is mediated by phosphorylation of the ERK1/2 signal^29^. In cardiac surgery patients at high risk for postoperative AKI, increased HMGB1 and Sema5b levels after rIPC were associated with renal protection after surgery^16^. Previous study demonstrated that the cardio-protection effect and mechanism of early and late rIPC were different^30^, while both early and late rIPC strategy significantly improved kidney function^15^, ^16^. Mechanism studies on specific molecules involved in the treatment of rIPC for AKI remained limited, which hinders the clinical application and protocol optimization of rIPC. Therefore, in this study, we utilized different AKI models (cisplatin, LPS, and IRI) to observe the renoprotective effects of different rIPC strategies *in vivo* and *in vitro* and to elucidate the potential signaling pathways involved in rIPC.

The NOX family is a crucial source of ROS and plays a fundamental role in redox signaling regulation. NOX4, the most widely expressed isoform, is a constitutive enzyme found in the kidney^7^ and plays a pivotal role in the modulation of oxidative stress, mitochondrial dysfunction, and the inflammatory response^8^, ^31^, ^32^. Our previous study demonstrated that genetic or pharmacological inhibition of NOX4 protected kidney function by preserving mitochondrial function and suppressing inflammation in septic AKI^10^, ^33^. RNA sequencing further revealed that NOX4 gene expression was significantly attenuated in rIPC-treated AKI mice. Therefore, we speculated that the protective effect of rIPC against AKI might be mediated by NOX4 signaling. Our study revealed consistent protective effects of rIPC across different AKI models. These protective effects were further enhanced or mitigated through NOX4 knockout/silencing or overexpression, respectively, confirming the mediating role of NOX4 during rIPC intervention in AKI. Interestingly, Dénes et al reported that rIPC significantly decreased IR-induced hepatic NOX2 expression but did not affect NOX4 expression^13^. This discrepancy may be attributed to several factors. First, the study by Dénes et al. primarily focused on the protection against leukocyte-endothelial cell interactions, and NOX2, rather than NOX4, is the predominant NOX homolog in liver immune cells, such as Kupffer cells and neutrophils^34^. In contrast, our study centered on tubular epithelial cells, where NOX4 is the dominant NOX homolog. Second, apart from organ/tissue specificity, differences in the animal species (rat vs. mouse) and rIPC protocols further contribute to the heterogeneity between the 2 studies. In fact, some other studies have reported that NOX4 activation contributes to hepatocyte injury, and its inhibition alleviates liver damage^35^, ^36^, which aligns with the observations in the kidney. We employed three distinct AKI models (IRI, LPS, and cisplatin) in our experiments, all of which consistently demonstrated trends in NOX4 expression, thereby enhancing the robustness of our results. Future studies are warranted to further explore the impact of rIPC on different NOX isoforms in various organs.

A reduction in oxidative stress is one of the potential mechanisms through which rIPC protects against AKI. Cells may undergo necrosis or programmed cell death if the levels of ROS, which primarily originate from mitochondria, are not properly regulated by antioxidative enzymes such as catalase or superoxide dismutase (SOD). An earlier RCT involving 60 patients who underwent angiography showed that rIPC alleviated the incidence of contrast-induced AKI, which might be mediated by decreasing oxidative stress^37^. Mechanistically, the phosphorylation of glycogen synthase kinase-3β (GSK-3β) has been shown to mediate the protective effect of rIPC against contrast-induced AKI by inhibiting mitochondrial permeability and reducing oxidative stress and tubular apoptosis^38^, ^39^. In cisplatin-induced AKI, Zhang and colleagues demonstrated that elevation of miR-144 via rIPC activated PTEN/AKT signaling to achieve an antiapoptotic effect^15^. In the present study, we observed that rIPC was associated with the amelioration of NOX/ROS overproduction and the preservation of mitochondrial dynamics/function in multiple AKI models, while the overexpression of NOX4 abrogated these protective effects. As a certain amount of ROS is a "necessary evil" for maintaining mitochondrial function and other cell signaling pathways^40^, the balance between antioxidative enzymes such as SOD and ROS source enzymes such as NOX4 might need to be accurately regulated.

Mitophagy, the process of specifically engulfing and sequestering aged and damaged mitochondria into lysosomes, is recognized as a genuine approach for mitigating the production of ROS^41^. Various studies have demonstrated the important role of mitophagy in AKI through different pathways, such as the SIRT3-, BNIP3- and PINK1-mediated mitophagy pathways^42^-^44^. A recent study showed that NOX4-Nrf2 redox imbalance contributes to mitophagy disturbance and kidney dysfunction in cisplatin-AKI^45^. In IRI-AKI, IPC confers resistance to AKI through Fundc1-dependent mitophagy through the reconciliation of mitochondrial fission^46^. The relationship between mitochondria oxidative stress and mitophagy are interwoven^47^. In our study, rIPC substantially ameliorated mitochondrial oxidative stress and subsequently corrected the disturbance of mitophagy, as reflected by the upregulation of p62, LC3B and PINK1. Classic mitophagy activation is manifested by the upregulation of LC3 and the downregulation of p62, while upregulation of both of them might indicate a state of aberrant autophagic flux characterized by increased autophagy induction and altered autophagosome fusion. Our results are consistent with some previous studies where mitophagy induction was decreased or autophagosome fusion was reactivated following effective treatment^48^-^50^. Our study indicates that rIPC might executes its renal protection effect by inhibiting upstream ROS production through inhibiting NOX4, but whether rIPC has a direct impact targeting mitophagy or that the changes in mitophagy is merely a reaction to ameliorated oxidative stress following rIPC treatment warrants further investigation. It's worth mentioning that the conversion from LC3BI to LC3BII, and the degeneration of p62 through lysosome digestion are time-phase dependent dynamic processes^51^, ^52^. Future studies are needed to clarify the details in accurate regulation of autophagic flux during AKI and the impact of rIPC on it.

Ferroptosis is a form of cell death that results from the excessive accumulation of lipid ROS caused by GPX4 inactivation and iron accumulation^53^. Our research revealed that ACSL4 was highly expressed and that GPX4 was significantly reduced during AKI, while rIPC reversed these abnormalities in the expression of these key regulators and protected against ferroptosis through the inhibition of NOX4 signaling. During AKI, oxidative stress led to tubular ferroptosis as well as mitophagy disturbance, which were both ameliorated following rIPC treatment targeting NOX4-ROS pathway. On the other hand, an interconnection between mitophagy and ferroptosis during AKI has also been reported. As a survival mechanism that removes damaged mitochondria and related ROS, mitophagy prevents cell death by modulating genes associated with ferroptosis^47^. *Lin* et al. reported that BNIP3-mediated and PINK1-mediated mitophagy protects against cisplatin-induced renal tubular epithelial cell ferroptosis through the ROS/HO1/GPX4 axis^54^. A more profound understanding of the interplay of these pathways would help determine how rIPC is translated into clinical benefits and identify employable rIPC strategies.

Although animal and clinical research into rIPC has been ongoing for three decades, there are still many unknowns regarding protocol implementation^55^. As in our research, the classic protocol of one treatment of four cycles of 5 minutes of ischemia followed by 5 minutes of reperfusion has been widely used in many studies, and its protection could last up to 3 to 4 days^56^. According to a study evaluating the IRI-AKI-to-CKD transition, repeated episodes of IPC, rather than one episode of IPC, resulted in long-term renal protection along with HO-1 overexpression and an increase in M2 macrophages^57^, but its applicability is limited by the complexity of the protocol. In addition to the rIPC protocol, the time interval between rIPC and AKI insult is another technical detail that remains unclear. In our study, we used two times intervals, 15 minutes and 6 hours, both of which achieved satisfactory renoprotection. However, “one-rIPC-treatment” strategy might not be able to cover late-onset tissue injury occurring weeks or months later. Given the potential limited protective time window of one-rIPC treatment, its long-term therapeutic effect needs to be clarified.

The strengths and limitations of our study are as follows. First, despite being the first to confirm the intermediating role of NOX4 in the protection of rIPC against AKI, the molecules responsible for carrying remote messages to regulate kidney NOX4 have not been identified. Exosomes could be crucial messengers in the renoprotective effects of rIPC^17^, which might help to elucidate the mechanism of NOX4 modulation and is part of our future work. Second, this study was advantageous in employing three AKI models—cisplatin, LPS and IRI-AKI—to address the heterogeneity across different AKI etiologies and ensure the robustness of our findings. However, given the complexity of AKI etiology, some AKI models, such as cecal ligation and puncture-induced-AKI, contrast-induced AKI, were not included and warranted further investigation. Third, we used the classic rIPC protocol, which was derived from the first study on IPC conducted more than 30 years ago^22^. AKI was induced at 15 minutes and 6 hours after rIPC, and the mice were harvested at different timepoint depending on different AKI models. The long-term protective effects of this rIPC protocol and other IPC strategies, such as chronic rIPC or repeated rIPC, await validation in the future. Fourth, in our *in vitro* experiment, we employed CCCP to mimic “chemical” ischemic-preconditioning through mitochondrial uncoupling, which enables us to focus on the mechanism at mitochondrial level. However, hypoxia-reoxygenation model might still be needed to reflect rIPC comprehensively in future studies.

## Conclusion

NOX4 was upregulated in AKI, while rIPC effectively reduced NOX4 expression. rIPC significantly attenuated oxidative stress, protected against mitochondrial injury and ameliorated tubular epithelial ferroptosis during AKI. Genetic or pharmacological inhibition of NOX4 mimicked the renoprotective effect of rIPC, while NOX4 knockout/silencing or overexpression accordingly enhanced or mitigated the therapeutic efficacy of rIPC. Therefore, rIPC may protect tubular epithelial mitochondrial function and attenuate ferroptosis in AKI by inhibiting NOX4-ROS signaling. Future studies are needed to elucidate the mechanism of remote regulation of kidney NOX4 and its messenger molecules. NOX4 might be used as a biomarker for monitoring the biological effects of rIPC, which enables us to optimize the rIPC protocol and facilitate clinical application.

## Supplementary Material

Supplementary methods, figures and tables.

## Abbreviations

rIPC: Remote ischemic preconditioning
CP: cisplatin
LPS: lipopolysaccharides
IRI: ischemia/reperfusion injury
sCr: serum creatinine
BUN: blood urea nitrogen
Lcn2: (neutrophil gelatinase-associated lipocalin, NGAL)
Havcr1: (kidney injury molecule 1, KIM1)
AKI: Acute kidney injury
NOX4tecko: Tubular epithelial cell-specific NOX4 knockout
siNOX4: NOX4 gene was silenced
Ad-NOX4: overexpressed by adenoviruses
ROS: reactive oxygen species
CCCP: carbonyl cyanide 3-chlorophenylhydrazone
H&E: hematoxylin and eosin
GSH: glutathione
