# Supplementary material for: Remote Ischemic Preconditioning Attenuates Mitochondrial Dysfunction and Ferroptosis of Tubular Epithelial Cells by Inhibiting NOX4-ROS Signaling in Acute Kidney Injury
Source: Int J Biol Sci. 2025 Feb 26;21(5):2313–29. doi: 10.7150/ijbs.105667 (PMC11900797; doi:10.7150/ijbs.105667)
Supplement: Supplementary file 1 — Supplementary methods, figures and tables. [file ijbsv21p2313s1.pdf]

Remote Ischemic Preconditioning Attenuates Mitochondrial Dysfunction and Ferroptosis of Tubular Epithelial Cells by Inhibiting NOX4-ROS Signaling in Acute Kidney Injury

This PDF file includes:

1. Supplementary Methods

Animals and treatments, Cell siRNA transfection, Adenoviral transfection, and H&E staining and evaluation.

2. Supplementary Figure

Supplementary Figure S1. Generation of renal tubular epithelial cell-specific (RTEC-specific) NOX4 knockout mice.

Supplementary Figure S2. Mating strategy to generate NOX4 conditional knockout in mouse RTECs.

Supplementary Figure S3. Pre-experiments on optimal dosage of CCCP in TCMK-1 cells.

Supplementary Figure S4. rIPC attenuates inflammation, mitochondrial malfunction and ferroptosis in LPS-induced AKI.

Supplementary Figure S5. rIPC attenuates inflammation, mitochondrial malfunction and ferroptosis in IRI-induced AKI.

Supplementary Figure S6. The expression and characteristics of NOX4 in healthy human adult and AKI patients' kidney single cells.

Supplementary Figure S7. rIPC reverses the upregulation of NOX4 in LPS and IRI-induced AKI.

Supplementary Figure S8. Pre-experiments on optimal dosage and timing of NOX4 overexpression by adenovirus in TCMK-1 cells.

3. Supplementary Tables

Supplementary Table S1. Primary antibodies used in the experiments.

Supplementary Table S2. Sequences of the primers for RT-qPCR.

1. Supplementary Methods

Animals and treatments

6-8 weeks old male C57BL/6J mice were purchased from GemPharmatech, Chengdu, China. Lipopolysaccharide (LPS)-induced AKI was established by injecting LPS (10mg/kg) intraperitoneally. Ischemic-reperfusion injury (IRI)-induced AKI was established by clamping both sides of the renal pedicles for 30 min. Additionally, GKT137831 was dissolved with 2% DMSO, 2% Tween80, 30% PEG300, and 66%ddHO.

$NOX4^{flox/flox}$ ($NOX4^{fl/fl}$) and renal tubular epithelial cell-specific (TEC-specific) conditional NOX4 knockout (Cdh16-Cre+$NOX4^{fl/fl}$, $NOX4^{tecKO}$) C57BL/6J mice were purchased from GemPharmatech, Nanjing, China. NOX4 knockout target site, sequence details and identification of the genotypes of mice were shown in **Fig S1A**. The construction of $NOX4^{fl/fl}$ mice is based on CRISPR/Cas9-stimulated homologous recombination. Briefly, exon 3 and exon 4 of the NOX4 gene were flanked by two LoxP elements. Two heterozygous recombinant embryonic stem cells clones screened by homologous recombination were identified and microinjected into blastocysts from C57BL/6J mice to generate floxed heterozygous mice ($NOX4^{flox/+}$). $NOX4^{flox/+}$ mice were then inbred to obtain homozygous NOX4-floxed mice ($NOX4^{fl/fl}$). To generate $NOX4^{tecKO}$ mice, $NOX4^{fl/fl}$ mice were crossed with Cdh16-Cre mice. The genotype of $NOX4^{tecKO}$ mice was confirmed by PCR assay using specific primers (**Fig S1B**). Littermates carried the $NOX4^{fl/fl}$ transgene were used as controls. Mating strategy to generate NOX4 conditional knockout in mouse RTECs was provided in **Figure S2**.

Cell siRNA transfection

TCMK-1 cells were transiently transfected with siRNAs and were performed with transfection reagent Lipofectamine™ 2000 Reagent (Invitrogen, CA, USA) according to the manufacturer’s instructions. The sequences of NOX4 siRNA were listed as follows: sense 5’-CCAUUUGCAUCGAUACUAA-3’ and antisense 5’-UUAGUAUCGAUGCAAAUGG-3’ (RiboBio, Guangzhou, China).

Adenoviral transfection

Adenoviruses (Ad) harboring NOX4 (Ad-NOX4) and without NOX4 (Ad-Null) were purchased from Hanbio Tech, Shanghai, China. TCMK-1 cells were transfected with Ad-NOX4 and Ad-Null according to the procedure for adenoviruses from Hanbio Tech, Shanghai, China. Ad-Null has no transgene and was used as a control for Ad-NOX4. 1×10^{10} PFU/ml, the titer of adenoviruses, was selected in this research.

H&E staining and evaluation

One-quarter of the kidney tissue was fixed in a 10% formaldehyde solution (50-00-0, Chron Chemicals, Chengdu, China) for paraffin embedding, and tissue sections (4 μm) were used for hematoxylin and eosin (H&E) staining. In a blinded manner, the images of sections were obtained by using the light microscopy at 200x and 400x magnifications. The damage of renal tubules was evaluated by the percentage of injured renal tubules and histological injury that was indicated by brush border lost, tubular dilation/flattening, tubular degeneration, tubular cast formation, and vacuolization. Ten fields of 400x magnification were evaluated and averaged. Tissue injury was scored on a scale of 0–4. In brief, the scores with 0, 1, 2, 3, and 4 was corresponding to 0, <25, 26–50, 51–75, and >76% of injured/damaged renal tubules, respectively.

2. Supplementary Figures

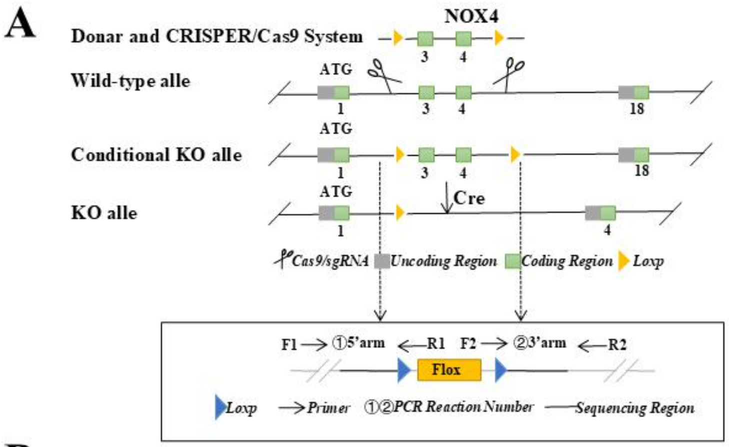

B

Primer Name	Sequence（5'-3'）	PCR size	Primer illustration
JS01996-Nox4-5wt-tF2	GTGCAGTTGCCTGTACCTGTAACC	Fl:364bp Wt:262bp	NOX4
JS01996-Nox4-5wt-tR2	GCAGAGCTACTGAAAGGAAAGGC		
T004820-F2	GGGCAGTCTGGTACTTCCAAGCT	KI:353bp	cdh16-Cre
T004820-R2	ACTGAGTGCCTACTAACCAGCACC		

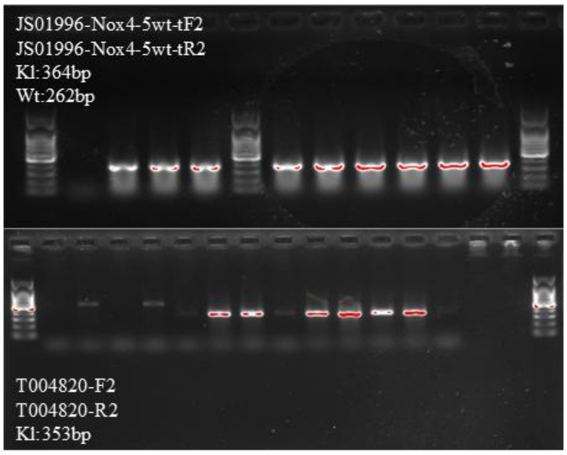

Supplementary Figure S1. Generation of renal tubular epithelial cell-specific (RTEC-specific) NOX4 knockout mice. (A) Schematic of NOX4$^{flox/flox}$ (NOX4$^{fl/fl}$) mice generation by CRISPR/Cas9-stimulated homologous recombination and design strategy of TEC-specific NOX4 KO (NOX4^{tecKO}) mice. (B) Successful transmission of Cdh16-Cre and NOX4$^{fl/fl}$ was confirmed by PCR genotyping.

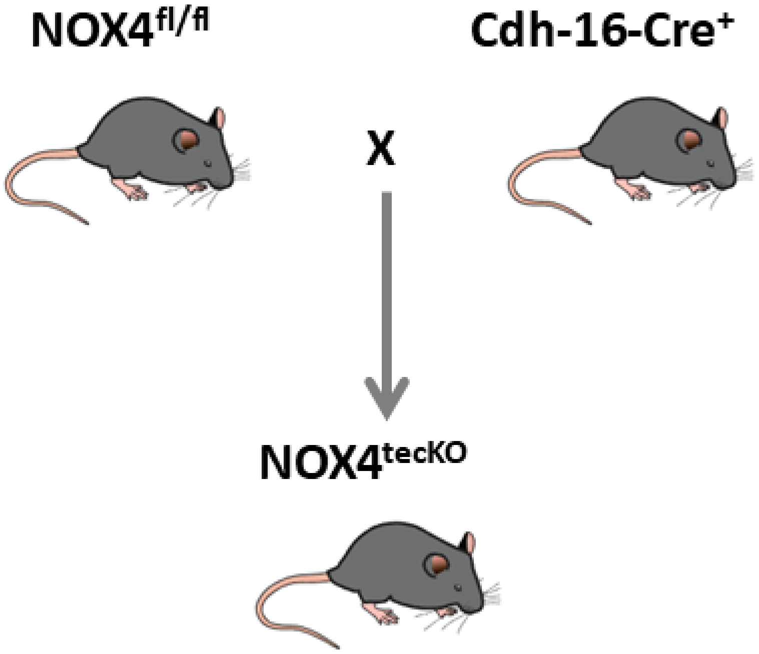

Supplementary Figure S2. Mating strategy to generate NOX4 conditional knockout in mouse RTECs.

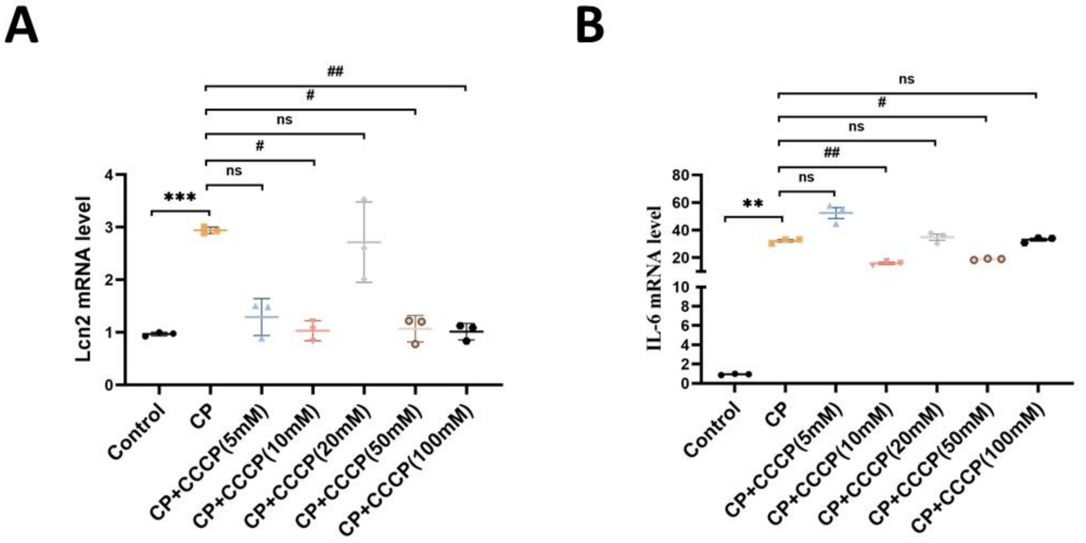

Supplementary Figure S3. Pre-experiments on optimal dosage of CCCP in TCMK-1 cells. (A) Lcn2 expression measured by RT−qPCR under different dosages of CCCP. (B) IL-6 expression measured by RT−qPCR under different dosages of CCCP. Data are presented as mean ± SD, n = 3. CCCP: carbonyl cyanide 3-chlorophenylhydrazone, CP: cisplatin, Lcn2: (neutrophil gelatinase-associated lipocalin, NGAL). *p<0.05, **p<0.01, ***p<0.001, ****p<0.0001, #p<0.05, ##p<0.01, ###p<0.001, ####p<0.0001, ns no significant.

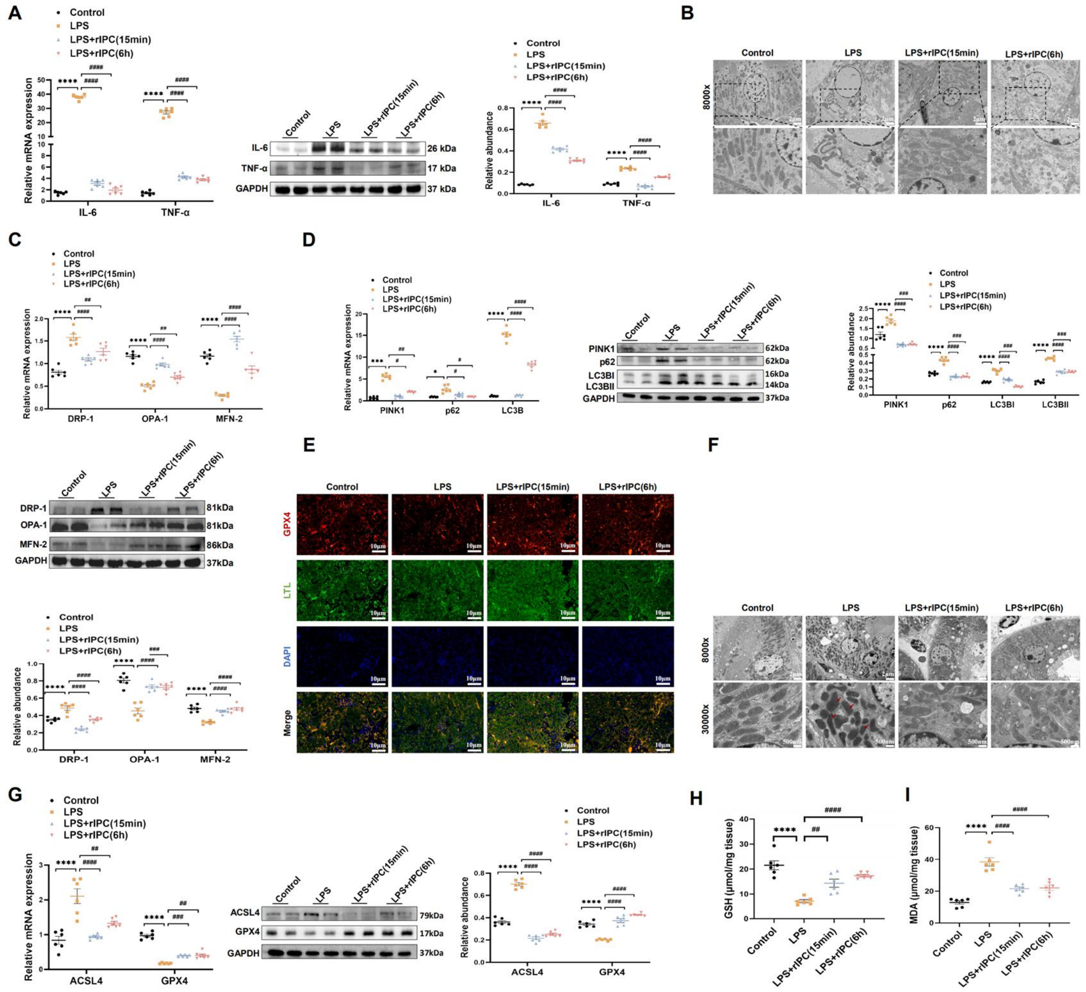

Supplementary Figure S4. rIPC attenuates inflammation, mitochondrial malfunction and ferroptosis in LPS-induced AKI. (A) IL-6 and TNF-α expression measured by RT-qPCR, western blot and quantified by densitometry in LPS-AKI mouse kidney. (B) The morphology of mitochondria examined by transmission electron microscope in LPS-AKI mouse kidney (8000x, scale bar = 2μm). (C) Mitochondrial dynamic regulatory molecules (DRP-1, OPA-1 and MFN-2) analyzed by RT-qPCR, western blot and quantified by densitometry in LPS-AKI mouse kidney. (D) Mitophagy level (PINK1, p62/SQSTM1 and LC3B) analyzed by RT-qPCR, western blot and quantified by densitometry in LPS-AKI mouse kidney. (E) Representative image of immunofluorescence staining of GPX4 in LPS-AKI mouse kidney (200x, scale bar = 10μm). (F) The morphological characteristic of ferroptosis under transmission electron microscope in LPS-AKI mouse kidney (8000x, scale bar = 2μm, 30000x, scale bar = 500nm). (G) Ferroptosis-related molecules (ACSL4 and GPX4) assessed by RT-qPCR, western bolt and quantified by densitometry in LPS-AKI mouse kidney. (H) The levels of GSH in LPS-AKI mouse kidney tissue. (I) The levels of MDA in LPS-AKI mouse kidney tissue. Data are presented as mean ± SD, n = 6. rIPC: remote ischemic preconditioning, LPS: lipopolysaccharides, GSH: glutathione. *p<0.05, **p<0.01, ***p<0.001, ****p<0.0001, #p<0.05, ##p<0.01, ###p<0.001, ####p<0.0001.

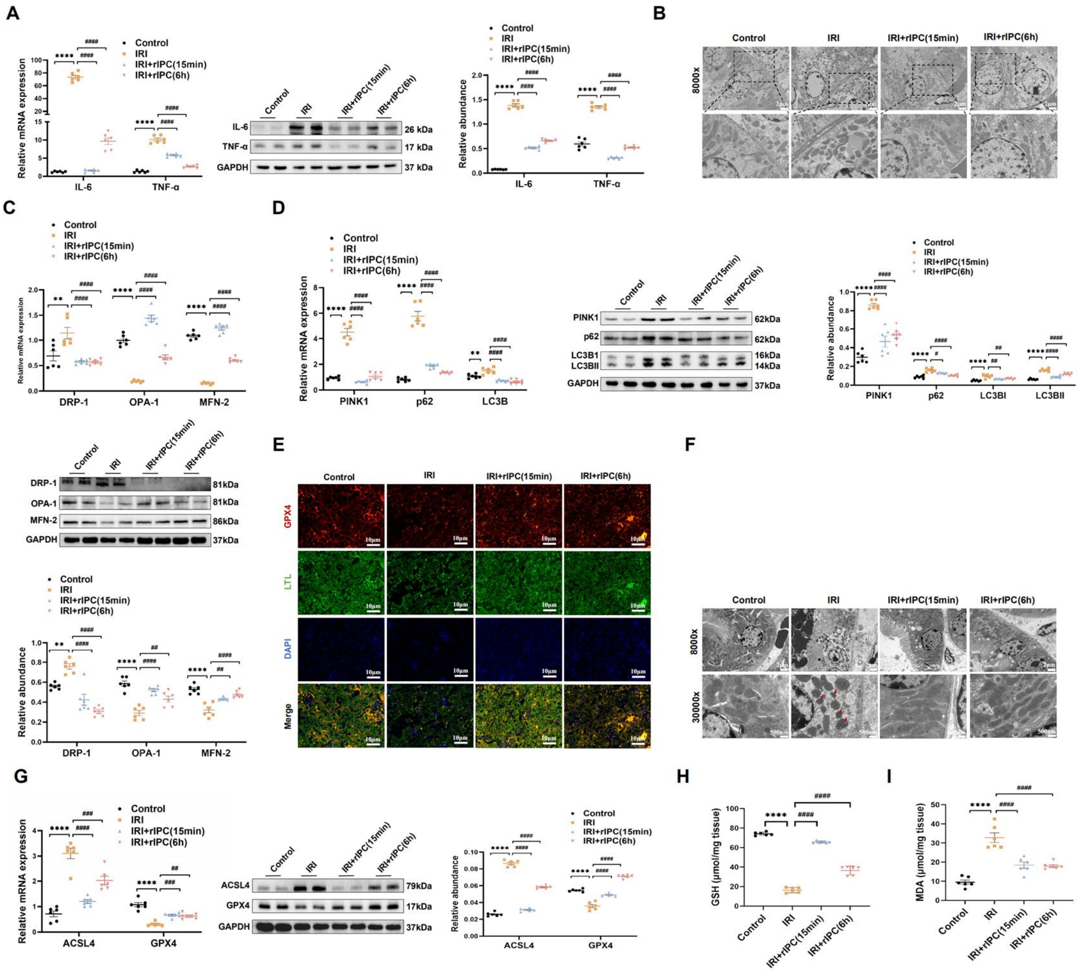

Supplementary Figure S5. rIPC attenuates inflammation, mitochondrial malfunction and ferroptosis in IRI-induced AKI. (A) IL-6 and TNF-α expression measured by RT-qPCR, western blot and quantified by densitometry in IRI-AKI mouse kidney. (B) The morphology of mitochondria examined by transmission electron microscope in IRI-AKI mouse kidney (8000x, scale bar = 2μm). (C) Mitochondrial dynamic regulatory molecules (DRP-1, OPA-1 and MFN-2) analyzed by RT-qPCR, western blot and quantified by densitometry in IRI-AKI mouse kidney. (D) Mitophagy level (PINK1, p62/SQSTM1 and LC3B) analyzed by RT-qPCR, western blot and quantified by densitometry in IRI-AKI mouse kidney. (E) Representative image of immunofluorescence staining of GPX4 in IRI-AKI mouse kidney (200x, scale bar = 10μm). (F) The morphological characteristic of ferroptosis under transmission electron microscope in IRI-AKI mouse kidney (8000x, scale bar = 2μm, 30000x, scale bar = 500nm). (G) Ferroptosis-related molecules (ACSL4 and GPX4) assessed by RT-qPCR, western bolt and quantified by densitometry in IRI-AKI mouse kidney. (H) The levels of GSH in IRI-AKI mouse kidney tissue. (I) The levels of MDA in IRI-AKI mouse kidney tissue. Data are presented as mean ± SD, n = 6. rIPC: remote ischemic preconditioning, IRI: ischemia/reperfusion injury, GSH: glutathione. *p<0.05, **p<0.01, ***p<0.001, ****p<0.0001, #p<0.05, ##p<0.01, ###p<0.001, ####p<0.0001.

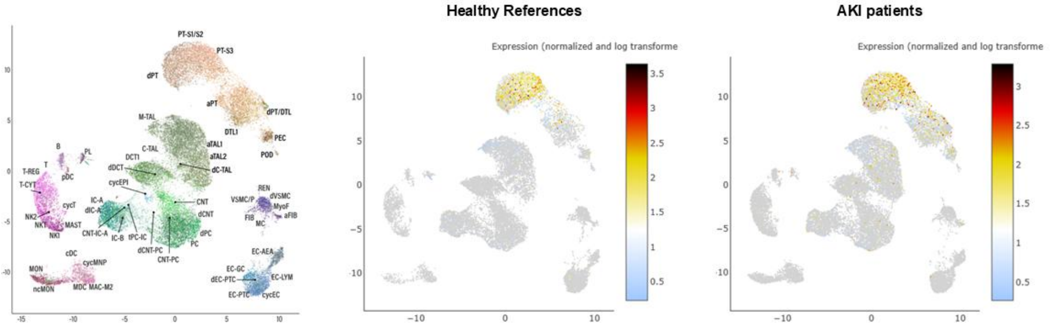

Supplementary Figure S6. The expression and characteristics of NOX4 in healthy human adult and AKI patients' kidney single cells. All open data were from The Kidney Precision Medicine Project (KPMP) (https://atlas.kpmp.org/). UMAP analysis presented that NOX4 was a few scattered within the kidney cells. aPT：Proximal Tubule Epithelial Cell (adaptive / maladaptive / repairing); dPT Proximal Tubule Epithelial Cell (degenerative); PT-S1/S2：Proximal Tubule Epithelial Cell Segment 1 / Segment 2; PT-S3：Proximal Tubule Epithelial Cell Segment 3; EC-AEA：Afferent / Efferent Arteriole Endothelial Cell; B, B Cell; cDC：Classical Dendritic Cell; CNT：Connecting Tubule Cell; dCNT：Connecting Tubule Cell (degenerative); CNT-IC-A：Connecting Tubule Intercalated Cell Type A; CNT-PC：Connecting Tubule Principal Cell; dCNT-PC：Connecting Tubule Principal Cell (degenerative); C-TAL：Cortical Thick Ascending Limb Cell; dC-TAL：Cortical Thick Ascending Limb Cell (degenerative); T-CYT：Cytotoxic T Cell; DTL1：Descending Thin Limb Cell Type 1; dDCT：Distal Convoluted Tubule Cell (degenerative); DCT1：Distal Convoluted Tubule Cell Type 1; cycEC：Endothelial Cell (cycling); cycEPI：Epithelial Cell (cycling); FIB：Fibroblast; aFIB：Fibroblast (adaptive /maladaptive / repairing); EC-GC：Glomerular Capillary Endothelial Cell; IC-A：Intercalated Cell Type A; dIC-A：Intercalated Cell Type A (degenerative); IC-B：Intercalated Cell Type B; EC-LYM：Lymphatic Endothelial Cell; MAC-M2：M2-Macrophage; MAST：Mast Cell; M-TAL：Medullary Thick Ascending Limb Cell; MC：Mesangial Cell; MON：Monocyte; MDC, Monocyte-derived Cell; cycMNP：Mononuclear Phagocyte (cycling);MyoF：Myofibroblast; NK1：Natural Killer Cell Type 1; NK2：Natural Killer Cell Type 2; NKT：Natural Killer T Cell; ncMON：Non-classical Monocyte; PEC：Parietal Epithelial Cell; EC-PTC：Peritubular Capillary Endothelial Cell; dEC-PTC：Peritubular Capillary Endothelial Cell (degenerative); PL：Plasma Cell; pDC：Plasmacytoid Dendritic Cell; POD：Podocyte; PC：Principal Cell; dPC：Principal Cell (degenerative); tPC-IC：Principal-Intercalated Cell (transitional); dPT/DTL：Proximal Tubule Epithelial Cell / Descending Thin Limb Cell (degenerative); T-REG：Regulatory T Cell; REN：Renin-positive Juxtaglomerular Granular Cell; T T Cell; cycT：T Cell (cycling2); aTAL1：Thick Ascending Limb Cell Cluster 1 (adaptive / maladaptive / repairing); aTAL2：Thick Ascending Limb Cell Cluster 2 (adaptive / maladaptive / repairing); dVSMC：Vascular Smooth Muscle Cell (degenerative); VSMC/P: Vascular Smooth Muscle Cell / Pericyte.

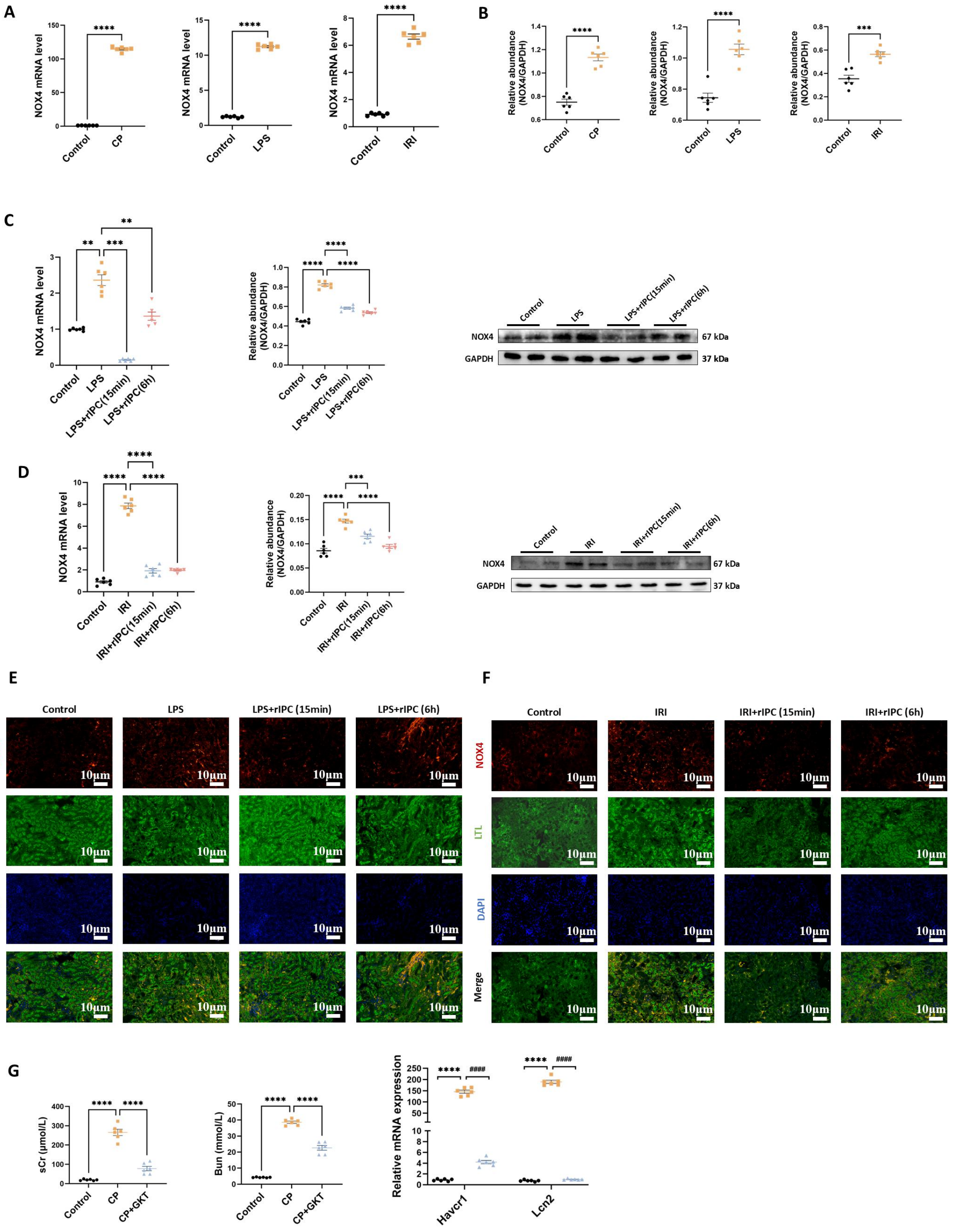

Supplementary Figure S7. rIPC reverses the upregulation of NOX4 in LPS and IRI-induced AKI. (A) The expression of NOX4 evaluated by RT-qPCR analysis in cisplatin, LPS and IRI-induced AKI models. (B) The protein expression of NOX4 by western blot quantified by densitometry in cisplatin, LPS and IRI-induced AKI models. (C) rIPC reverses the upregulation of NOX4 in LPS-induced AKI. (D) rIPC reverses the upregulation of NOX4 in IRI-induced AKI. (E) Representative image of immunofluorescence staining of NOX4 in kidney tissue sections in LPS-induced AKI (200x, scale bar = 10μm). (F) Representative image of immunofluorescence staining of NOX4 in kidney tissue sections in IRI-induced AKI (200x, scale bar = 10μm). (G) The levels of sCr, BUN in plasma and KIM-1 and NGAL in kidney were significantly reduced after GKT137831 treatment in CP-AKI. Data are presented as mean ± SD, n = 6. rIPC： remote ischemic

preconditioning, CP： cisplatin, LPS： lipopolysaccharides, IRI： ischemia/reperfusion injury, sCr： serum creatinine, BUN： blood urea nitrogen. *p<0.05, **p<0.01, ***p<0.001, ****p<0.0001, #p<0.05, ##p<0.01, ###p<0.001, ####p<0.0001.

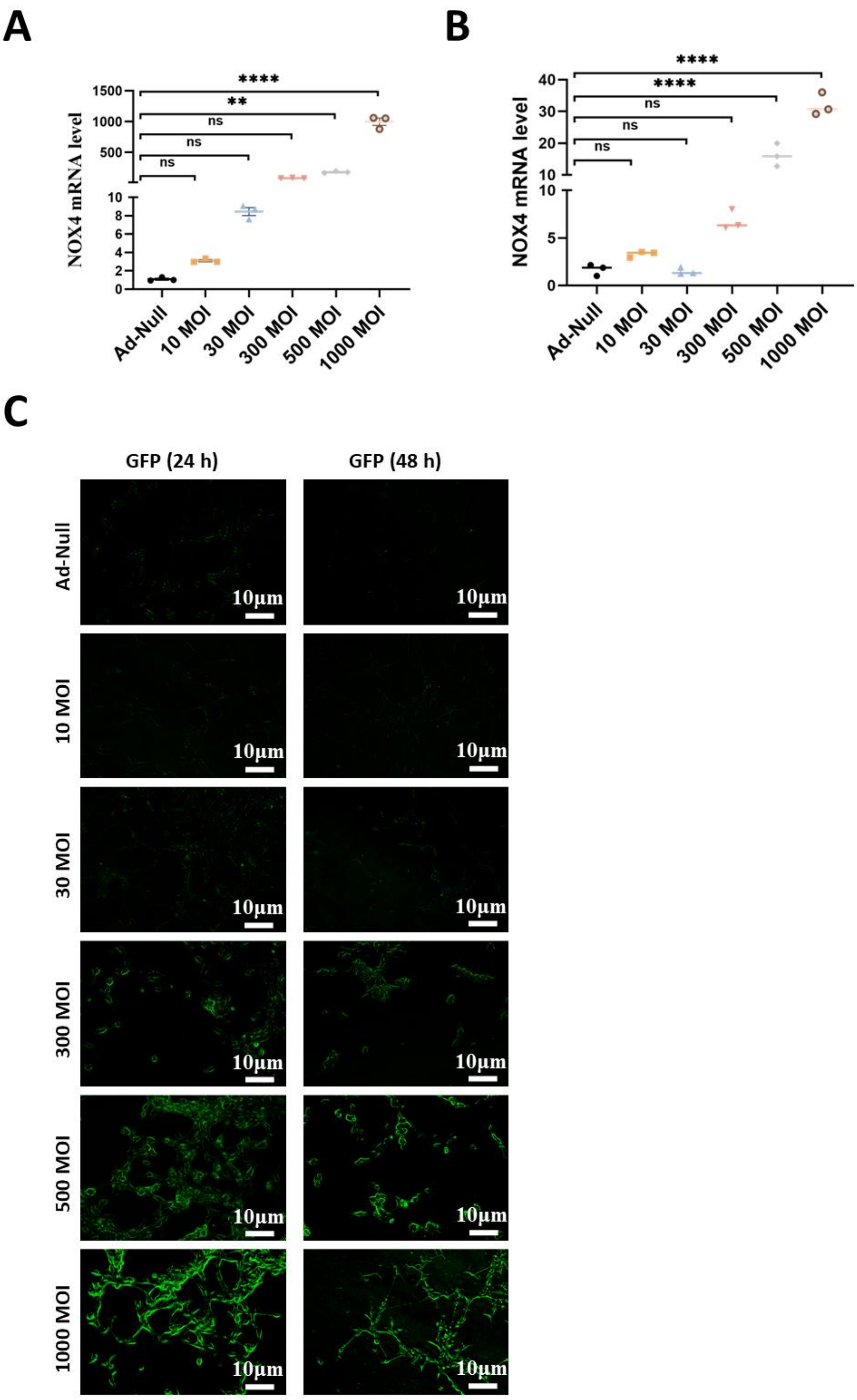

Supplementary Figure S8. Pre-experiments on optimal dosage and timing of NOX4 overexpression by adenovirus in TCMK-1 cells. (A) NOX4 expression measured by RT-qPCR under different dosages of Ad-NOX4 for 24 h in TCMK-1 cells. (B) NOX4 expression measured by RT-qPCR under different dosages of Ad-NOX4 for 48 h in TCMK-1 cells. (C) The expression of NOX4 under different dosages of Ad-NOX4 for 24 h and 48 h in TCMK-1 cells evaluated by fluorescence (200x, scale bar = 10 μm). Data are presented as mean ± SD. *p<0.05, **p<0.01, ***p<0.001, ****p<0.0001, ns no significant.

3. Supplementary Tables

Supplementary Table S1. Primary antibodies used in the experiments.

Name	Company	Catalog Number
Anti-NOX4	Abcam, MA, US	ab133303
Anti-IL-6	HuaAn Biotechnology, Hangzhou, China	EM170414
Anti-TNF-α	Affinity Biosciences, Changzhou, China	AF7014
Anti-DRP-1	Proteintech Group, Wuhan, China	12957-1-AP
Anti-OPA-1	Affinity Biosciences, Changzhou, China	DF8587
Anti-MFN-2	Proteintech Group, Wuhan, China	12186-1-AP
Anti-PINK1	Affinity Biosciences, Changzhou, China	DF7742
Anti-p62/SQSTM1	HuaAn Biotechnology, Hangzhou, China	EM0704
Anti-LC3B	HuaAn Biotechnology, Hangzhou, China	ET1707-65
Anti-ACSL4	HuaAn Biotechnology, Hangzhou, China	ET7111-43
Anti-GPX4	Abcam, MA, US	Ab125066
Anti-GAPDH	Zenbioscience, Chengdu, China	200306-7E4

Supplementary Table S2. Sequences of the primers for RT–qPCR.

Mouse gene	Sequence
F-NOX4	CAGATGTTGGGGCTAGGATTG
R-NOX4	GAGTGTTCGGCACATGGGTA
F-IL-6	ACAGAAGGAGTGGCTAAGGA
R-IL-6	AGGCATAACGCACTAGGTTT
F-TNF-α	ACCCTCACACTCAGATCATCTTC
R-TNF-α	TGGTGGTTTGCTACGACGT
F-NGAL	TGGCCCTGAGTGTCATGTG
R-NGAL	CTCTTGTAGCTCATAGATGGTGC
F-KIM1	ACATATCGTGGAATCACAACGAC
R-KIM1	ACTGCTCTTCTGATAGGTGACA
F-DRP-1	AGATGACCACCACTGTAGCC
R-DRP-1	AGCTTCCCCTTTCCCTGTTT
F-OPA-1	CTTCGTCTCTCCTCATCGGG
R-OPA-1	TGACATCCCACGCTGTACAG
F-MNF-2	GCATTCTTGTGGTCGGAGGAGTG
R-MFN-2	TGGTCCAGGTCAGTCGCTCATAG
F-PINK1	TGCAGTGCTGCTGTGTATGA
R-PINK1	GAACCTGCCGAGATGTTCCA
F-p62/SQSTM1	GCACCCCAATGTGATCTGC

R-p62/SQSTM1	CGCTACACAAGTCGTAGTCTGG
F-LC3B	ATTCGAGAGCAGCATCCAACC
R-LC3B	TGTCCGTTCACCAACAGGAAG
F-ACSL4	CTCACCATTATATTGCTGCCTGT
R-ACSL4	TCTCTTTGCCATAGCGTTTTTCT
F-GPX4	GCCTGGATAAGTACAGGGGTT
R-GPX4	CATGCAGATCGACTAGCTGAG
F-GAPDH	AGGTCGGTGAACGGATTG
R-GAPDH	TGTAGACCATGTAGTTGAGGTCA
